# Supplementary figures and images for: The structure of the human LACTB filament reveals the mechanisms of assembly and membrane binding
Source: PLoS Biol. 2022 Dec 19;20(12):e3001899. doi: 10.1371/journal.pbio.3001899 (PMC9815587; doi:10.1371/journal.pbio.3001899)

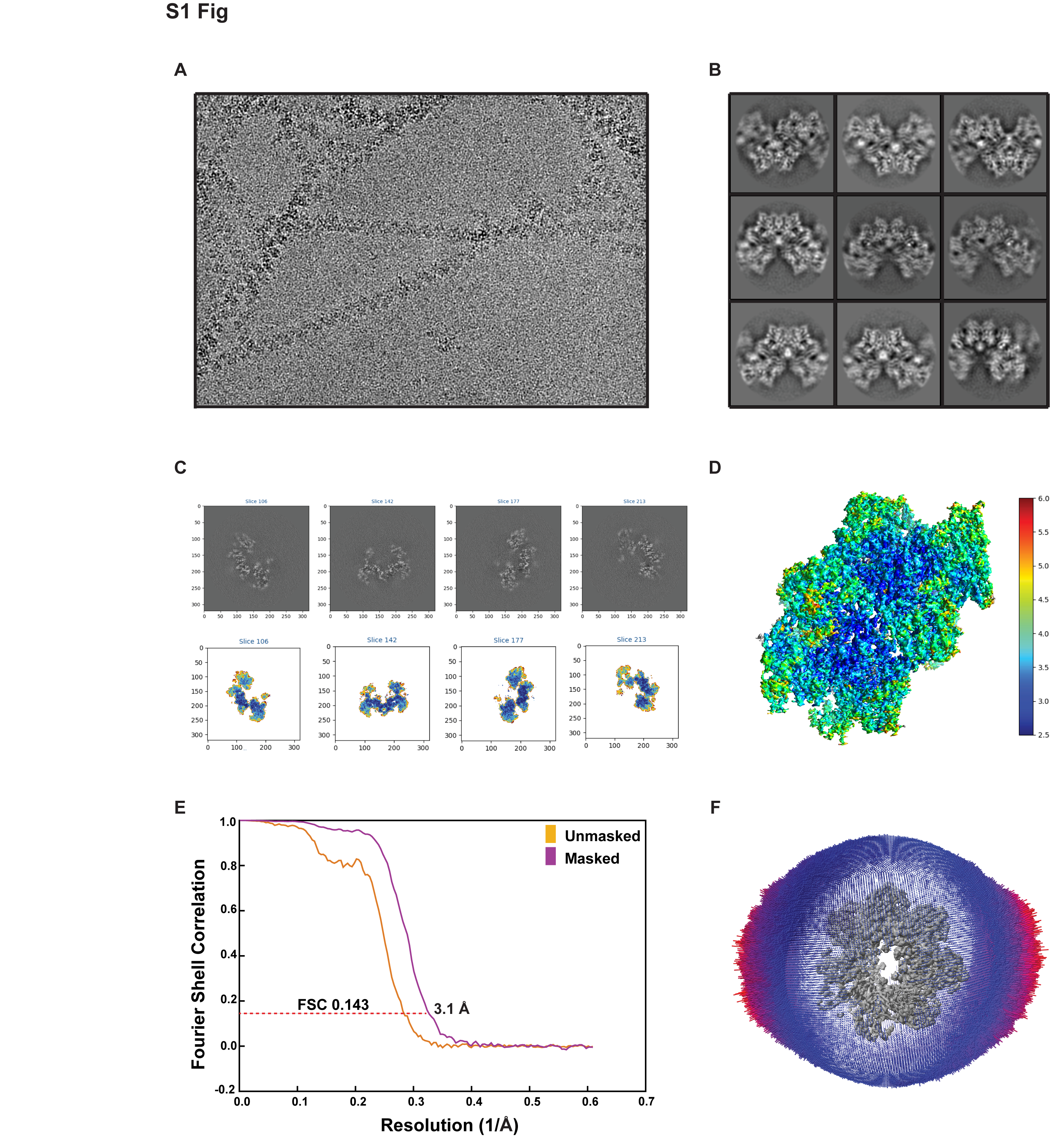

Supplement: S1 Fig — (A) A representative cryoEM micrograph and (B) 2D class averages of the human LACTB filament. (C) Slices through the unsharpened density map of the LACTB filament are shown in the top view. (D) CryoEM map of the human LACTB filament is colored according to the local resolution estimation calculated by ResMap. (E) Fourier Shell Correlation (FSC) curve shows 3.1 Å global resolution (masked in purple and unmasked in orange) with the gold standard criteria (FSC = 0.143). (F) Euler angle distribution of the segments used in the final 3D reconstruction. (TIF) [file pbio.3001899.s001.tif]

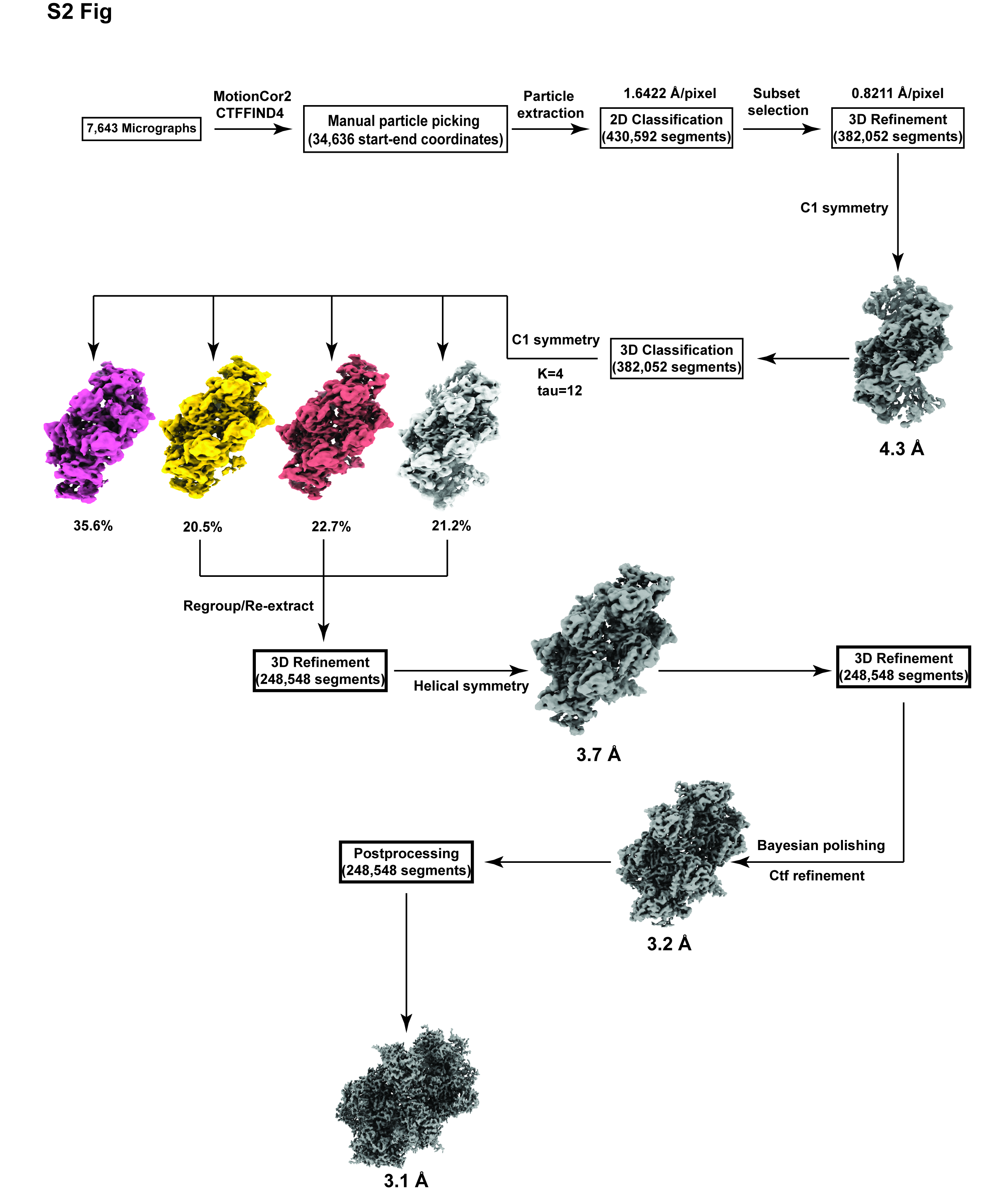

Supplement: S2 Fig — Flow chart summarizing cryoEM image-processing workflow, including 3D classification and 3D auto-refinement, CTF refinement, and Bayesian polishing. (TIF) [file pbio.3001899.s002.tif]

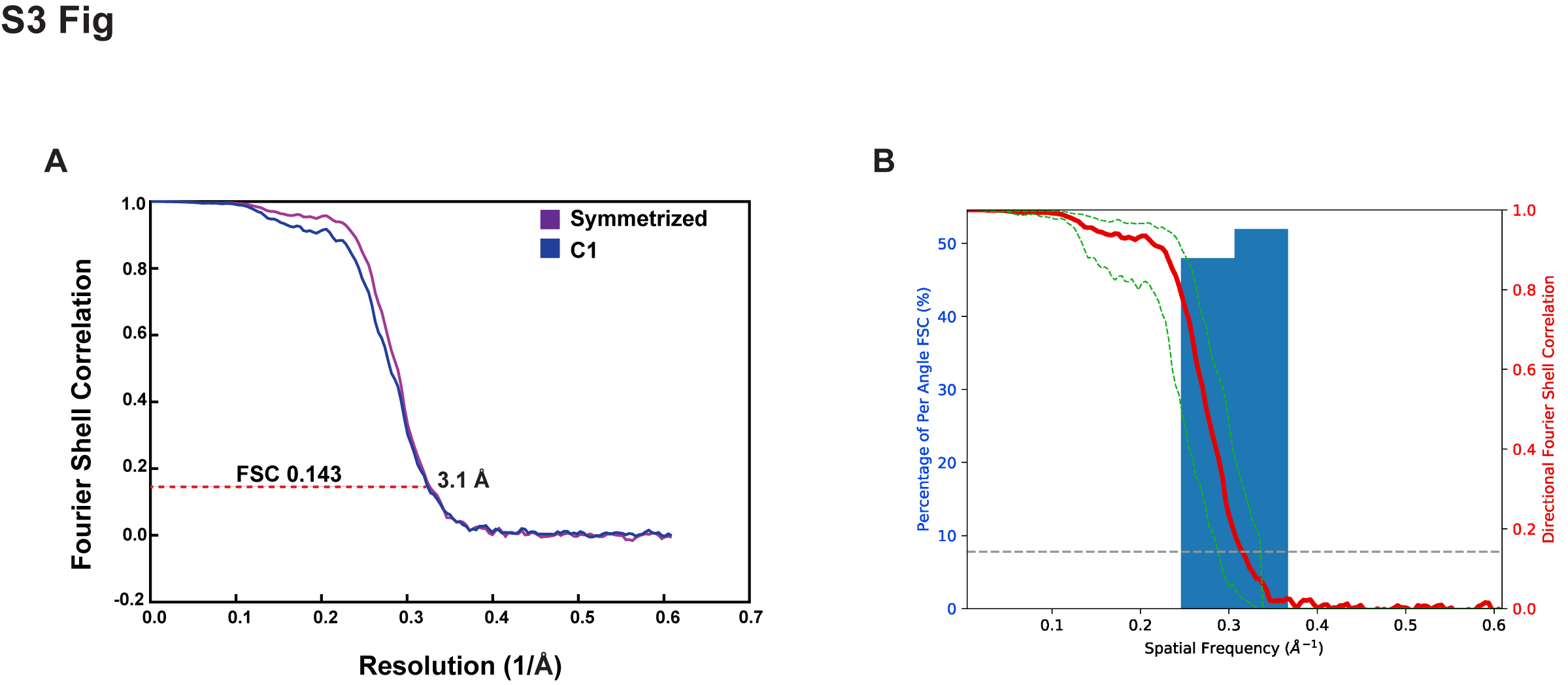

Supplement: S3 Fig — (A) Fourier Shell Correlation curves for the final helical symmetry imposed (purple line) and C1 symmetry (blue line) 3D reconstructions. (B) 3D Fourier Shell Correlation of the final cryoEM map. (TIF) [file pbio.3001899.s003.tif]

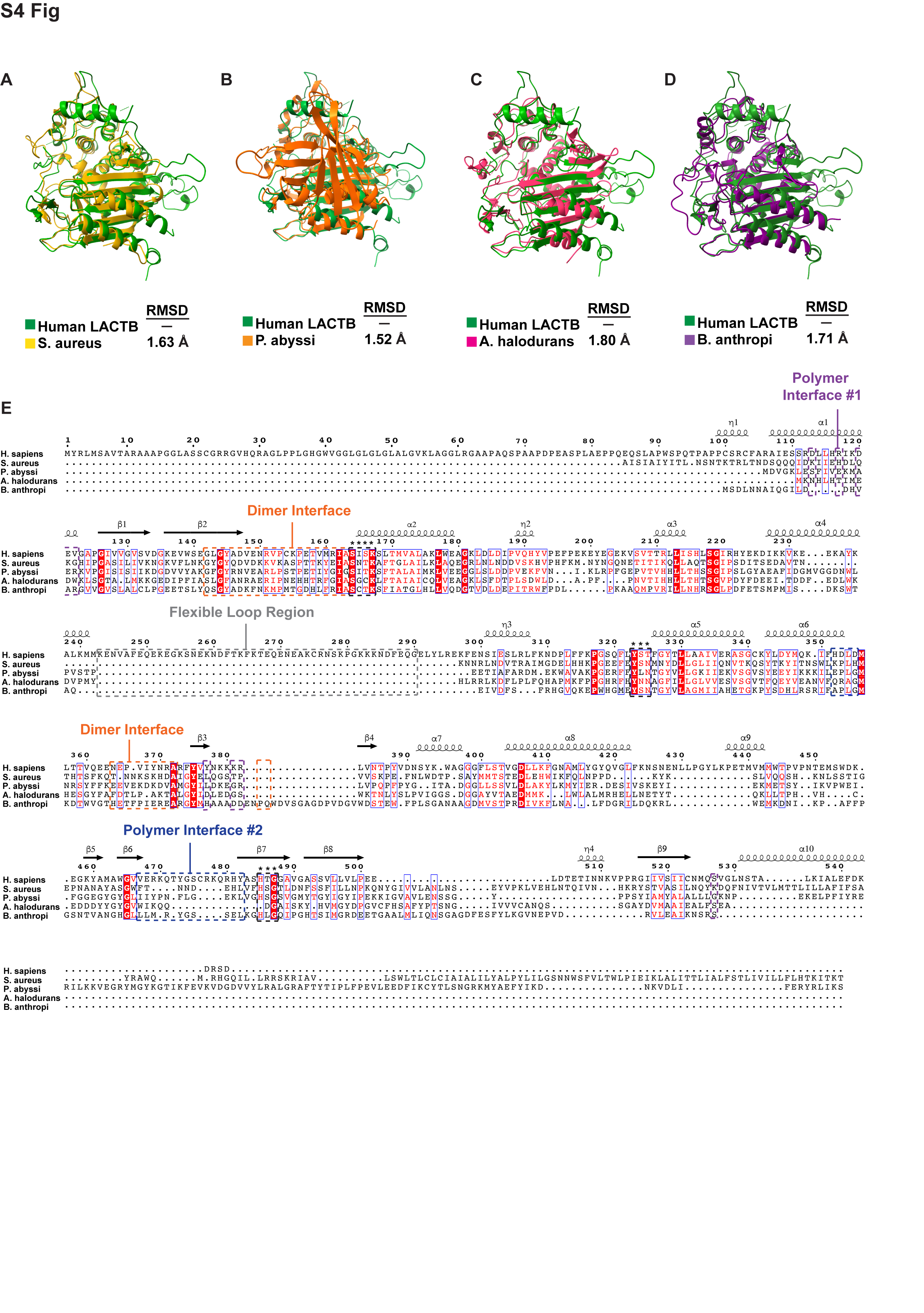

Supplement: S4 Fig — (A–D) Comparison of human LACTB monomer (green) with S. aureus ClbP protein (yellow, PDB ID: 4GDN), P. abyssi Pab87 peptidase (orange, PDB ID: 2QMI), A. halodurans penicillin-binding protein (pink, PDB ID: 3TG9), and B. anthropi D-amino-acid amidase (purple, PDB ID: 2EFU). The root-mean-square deviation of each structure relative to the LACTB monomer is shown at the bottom. While most β-strands align well between human and bacterial proteins, the surrounding helices and interface-forming loops show significant differences between these structures. (E) Sequence alignment of LACTB homologs and orthologs. Residues that form the dimerization interface and polymerization interfaces 1 and 2 are highlighted with orange, purple, and blue boxes, respectively. Catalytic site residues are highlighted with an asterisk. Secondary structure elements shown above the alignment are generated from the human LACTB structure. Sequences were aligned using Homo Sapiens LACTB (Uniprot ID: P83111), S. aureus ClbP (Uniprot ID: Q7A3Q5), P. abyssi Pab87 peptidase (Uniprot ID: Q9V2D6), A. halodurans penicillin-binding protein (Uniprot ID: Q9KAM0), and B. anthropi D-amino-acid amidase (Uniprot ID: Q9LCC8). (TIF) [file pbio.3001899.s004.tif]

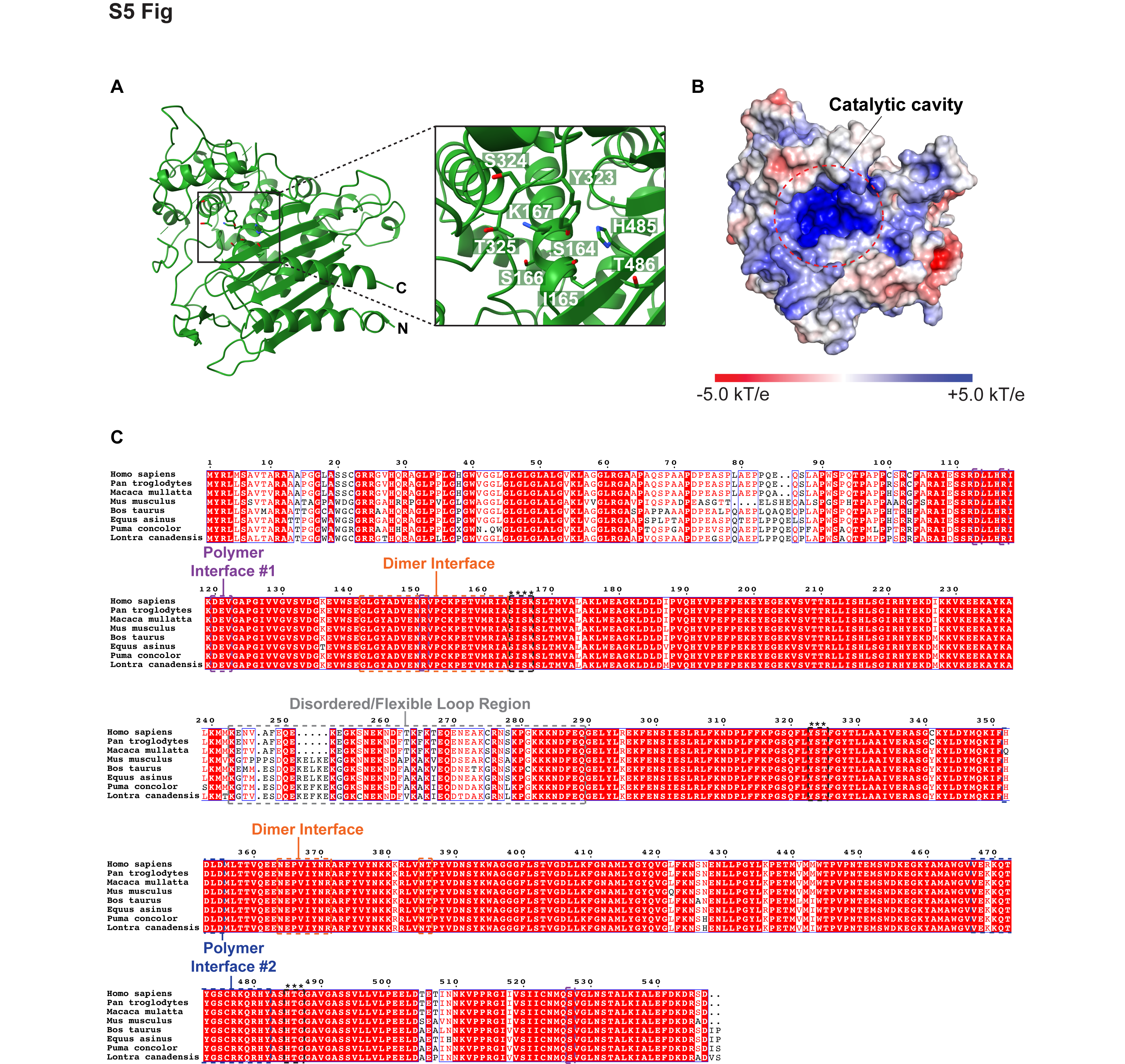

Supplement: S5 Fig — (A) Active site and ligand recognition of human LACTB. Ribbon diagram of a monomer is displayed in green and residues forming the catalytic site are shown as sticks. Boxed zoom images highlight the positions of the conserved residues that contribute to catalytic site formation. (B) Surface electrostatic potential representation of the LACTB monomer. Positive and negative electrostatic potentials are shown in blue and red, respectively. The substrate-binding cavity is highlighted with a red circle. (C) Sequence alignment of LACTB homologs. Residues that form the dimerization interface and polymerization interfaces 1 and 2 are highlighted with orange, purple, and blue boxes, respectively. Catalytic site residues are highlighted with an asterisk. Secondary structure elements shown above the alignment are generated from the human LACTB structure. LACTB sequences from Homo sapiens (human; Uniprot ID: P83111), Pan troglodytes (chimpanzee; Uniprot ID: K7CYM3), Macaca mulatta (rhesus macaque; Uniprot ID: F7EXQ6), Mus musculus (mouse; Uniprot ID: Q9EP89), Bos taurus (cow; Uniprot ID: P83095), Equus asinus (donkey; Uniprot ID: UPI001D03C0DD), Puma concolor (mountain lion; Uniprot ID: A0A6P6HFT1), and Lontra canadensis (river otter; Uniprot ID: UPI0013F34634) are aligned. (TIF) [file pbio.3001899.s005.tif]

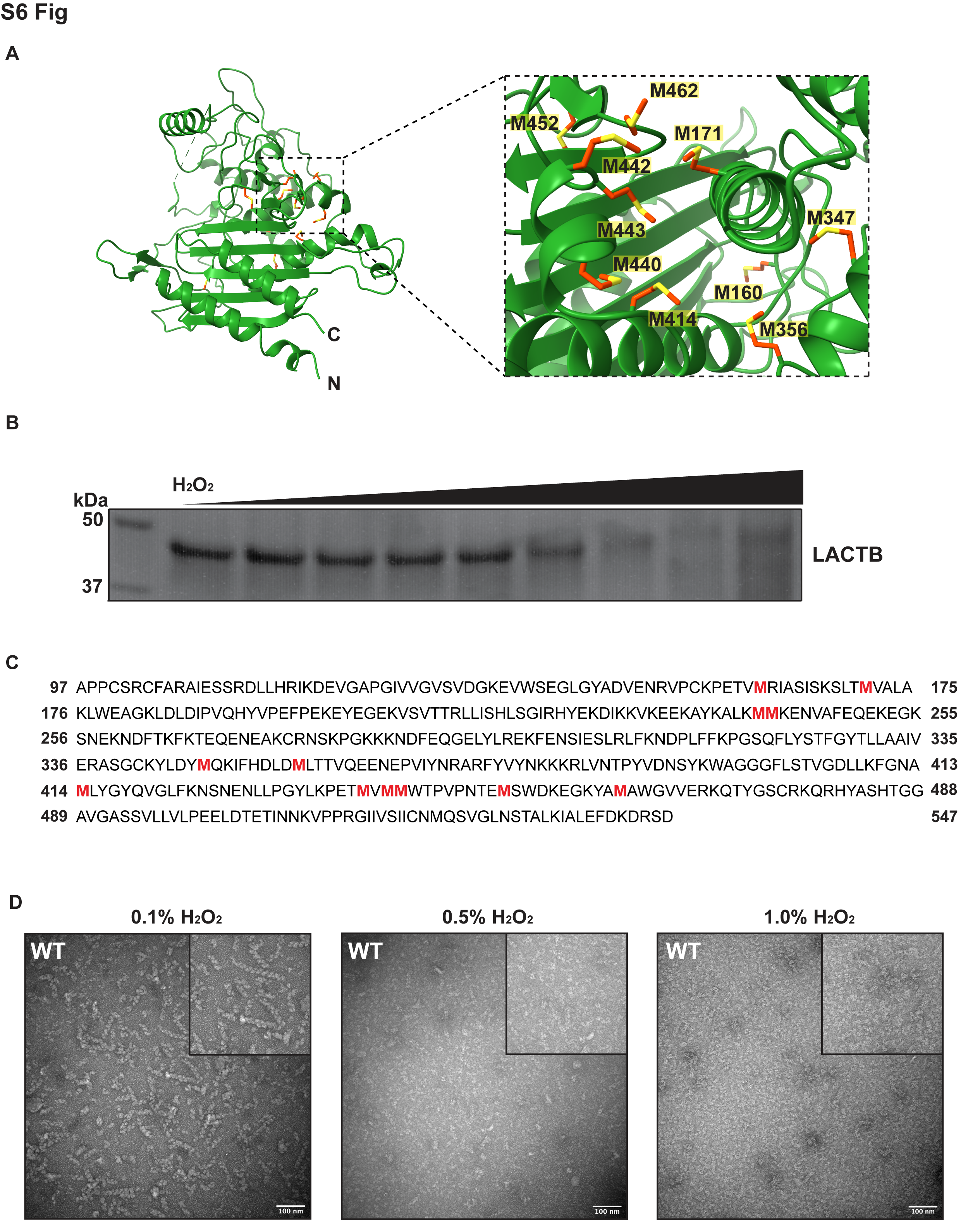

Supplement: S6 Fig — (A) Ribbon diagram of the LACTB monomer is displayed in green, and residues forming a methionine cluster are shown as sticks. Boxed zoomed in image highlights the positions of methionine residues that are colored orange and yellow. (B) Coomassie blue-stained SDS-PAGE gel of WT LACTB protein treated with increasing concentrations (0.1%, 0.5%, and 1.0%) of H2O2. Indicated concentrations of H2O2 were incubated with purified LACTB for 30 min before SDS-PAGE analysis. The left lane is the molecular weight standards, and their positions are indicated. (C) Sequence of WT LACTB construct (residues 97–547) with confirmation of methionine residues oxidized upon exposure to H2O2 (shown in red) by liquid chromatography with tandem mass spectrometry (LC-MS/MS). The underlying data for (C) is provided in S2 Data. (D) Representative negative-stain TEM micrographs of WT LACTB treated with varying concentrations of H2O2 for 30 min before grid preparation. Scale bars, 100 nm. (TIF) [file pbio.3001899.s006.tif]

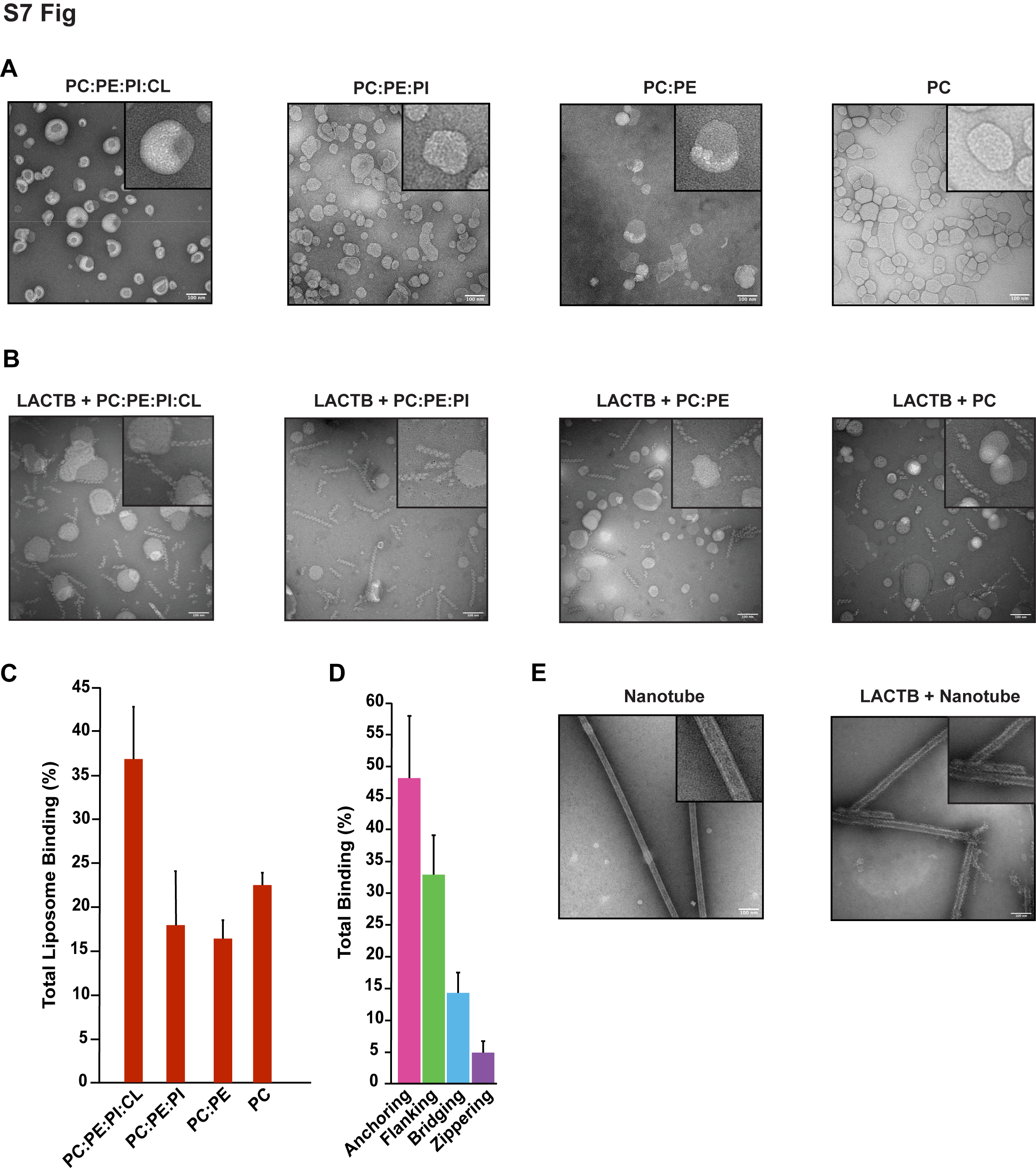

Supplement: S7 Fig — (A) Negative stain images of liposomes with different compositions. From left to right: PC:PE:PI:CL, PC:PE:PI, PC:PE, PC. (B) Negative stain images of WT LACTB in the presence of liposomes with different compositions in the same order as (A). (C) Total liposome binding activity of WT LACTB with liposomes of different compositions. Total liposome binding was quantified by counting 1,000 filaments from at least 3 independent reconstitution assays and calculating the total percentage of filament binding. Bars represent the mean of at least 3 independent reconstitution assays and error bars indicate the SEM. (D) Percentage of LACTB filaments bound to liposomes by interaction type. Bars represent the mean of at least 3 independent reconstitution assays and error bars indicate the SEM. (E) Negative stain images of empty nanotubes (left) and WT LACTB filaments in the presence of nanotubes (right). The source data for (C) and (D) are provided in sheets S7C and S7D Fig in S1 Data. (TIF) [file pbio.3001899.s007.tif]

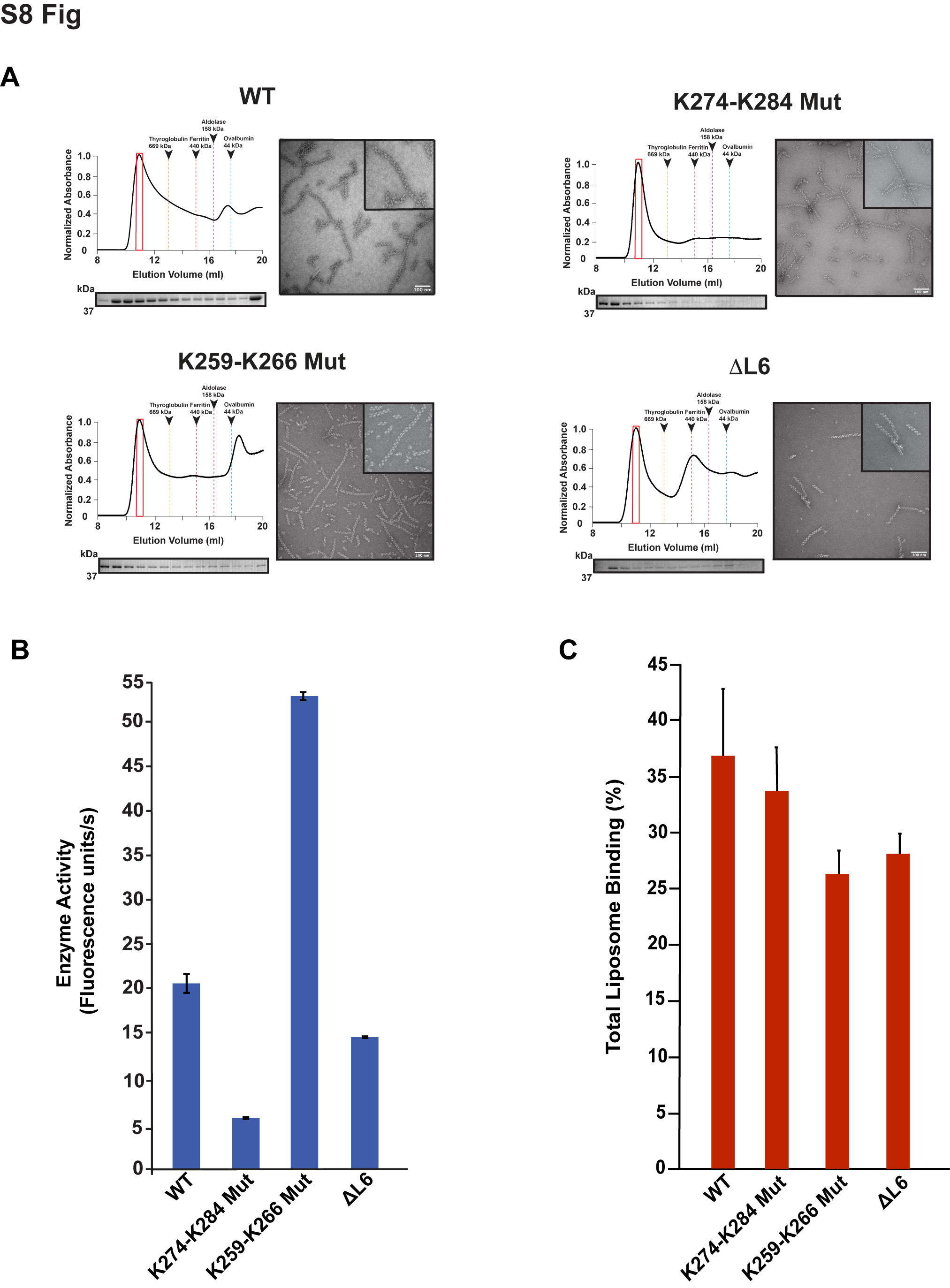

Supplement: S8 Fig — (A) Size-exclusion chromatography elution profiles of WT and mutant LACTB. SEC elution profiles begin with the void volume (V0) of the column. Fractions were analyzed by SDS-PAGE and peak fractions used in negative-stain analyses are highlighted in red boxes. Negative-stain TEM images demonstrate the polymerization activity of WT and mutant proteins. Scale bars, 100 nm. (B) Catalytic activity of key residues involved in lipid binding. Enzyme activity was determined in vitro by using a fluorescently labeled substrate and 3 independent experiments were performed for each sample. Bars represent the mean of at least 3 independent experiments and error bars represent the standard deviation. (C) Total liposome-binding activity of WT LACTB and key residues involved in lipid binding. Total liposome binding was quantified by counting 1,000 filaments from at least 3 independent reconstitution assays and calculating the total percentage of filament binding. Bars represent the mean of at least 3 independent reconstitution assays and error bars indicate the SEM. The underlying data in (B) and (C) are provided in sheets S8B and S8C Fig in S1 Data. (TIF) [file pbio.3001899.s008.tif]

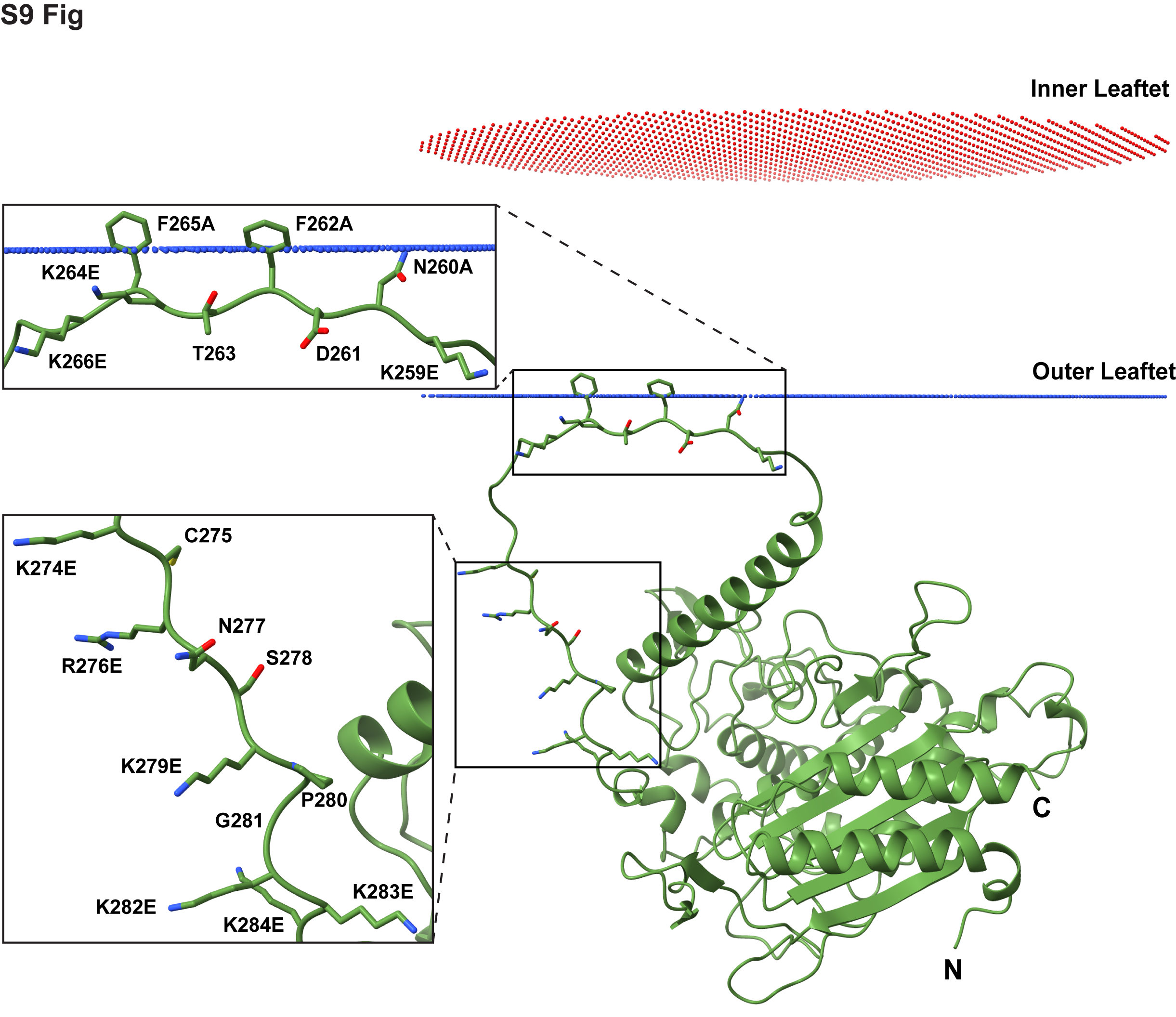

Supplement: S9 Fig — The monomeric structure of WT human LACTB was obtained from AlphaFold and the rotational and translation position of the protein was calculated in the presence of membranes, mimicking the lipid composition of the mitochondrial IM using the PPM Web Server. Boxed zoomed in images highlight the predicted structures of 259KNxFxKFK266 and 274KxRxxKxxKKK284 motifs within the flexible loop region (L6) of human LACTB. (TIF) [file pbio.3001899.s009.tif]

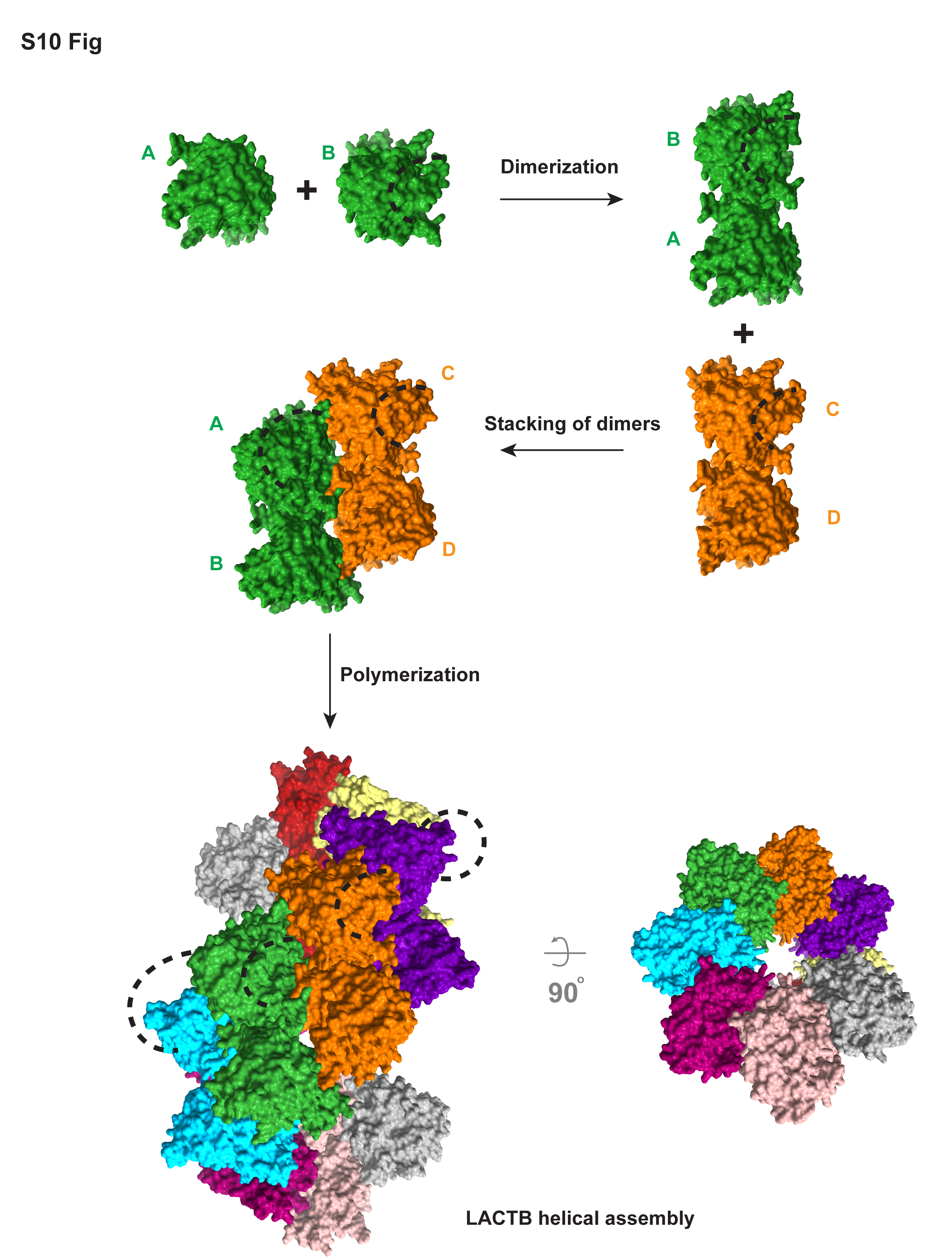

Supplement: S10 Fig — After being imported to mitochondrial IMS, LACTB protomers assemble into antiparallel dimers. Subsequently, LACTB dimers interact through 2 polymerization interfaces, which allow the stacking of antiparallel dimers in a helical fashion and lead to the polymerization into micron-scale helical assemblies. Upon filament formation, LACTB gains catalytic activity and filament elongation increases the catalytic efficiency of the enzyme. (TIF) [file pbio.3001899.s010.tif]
