# Supplementary material for: The structure of the human LACTB filament reveals the mechanisms of assembly and membrane binding
Source: PLoS Biol. 2022 Dec 19;20(12):e3001899. doi: 10.1371/journal.pbio.3001899 (PMC9815587; doi:10.1371/journal.pbio.3001899)
Supplement: S1 Table — Mutations associated with various cancers were compiled from the Catalogue of Somatic Mutations in Cancer (COSMIC), the Genome Aggregation Database (gnomAD), and The Cancer Genome Atlas Program (TCGA). (PDF) [file pbio.3001899.s011.pdf]

S1 Table | Human LACTB mutations associated with cancer

Protein change	Clinical classification	Cancer type
H116Q	Neutral	Thyroid carcinoma
E121K	Pathogenic	Breast
V148F	Pathogenic	Kidney
E149Q	Pathogenic	Esophagus
R151S, R151H	Likely pathogenic	Uterus
E363K	Pathogenic	Pancreatic
R371K	Pathogenic	Lung
A372T	Pathogenic	Uterus
K380N	Pathogenic	Cervical
R382L, R382C	Pathogenic	Oral, uterus
E457K	Pathogenic	Bladder
E468G	Pathogenic	Glioma
R469K	Pathogenic	Colon, breast
T472K, T472M	Neutral	Melanoma, colon
R480W, R480Q, R480L	Pathogenic	Lung, colon, liver
Y482H	Pathogenic	Melanoma
