# Supplementary material for: The structure of the human LACTB filament reveals the mechanisms of assembly and membrane binding
Source: PLoS Biol. 2022 Dec 19;20(12):e3001899. doi: 10.1371/journal.pbio.3001899 (PMC9815587; doi:10.1371/journal.pbio.3001899)

Figure 1C WT Captured by LAS 4000 Imager

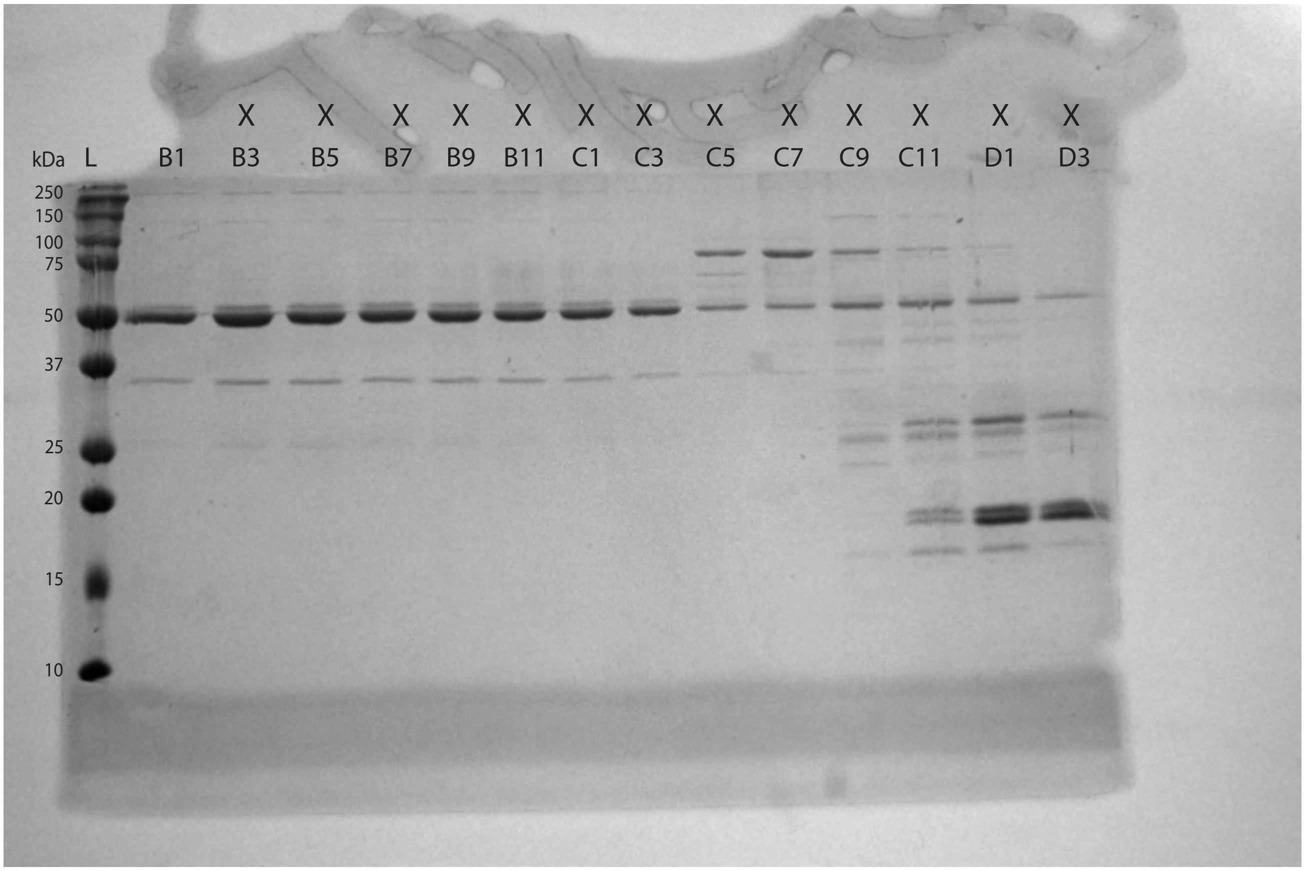

Figure 3C WT Captured by InGenius3

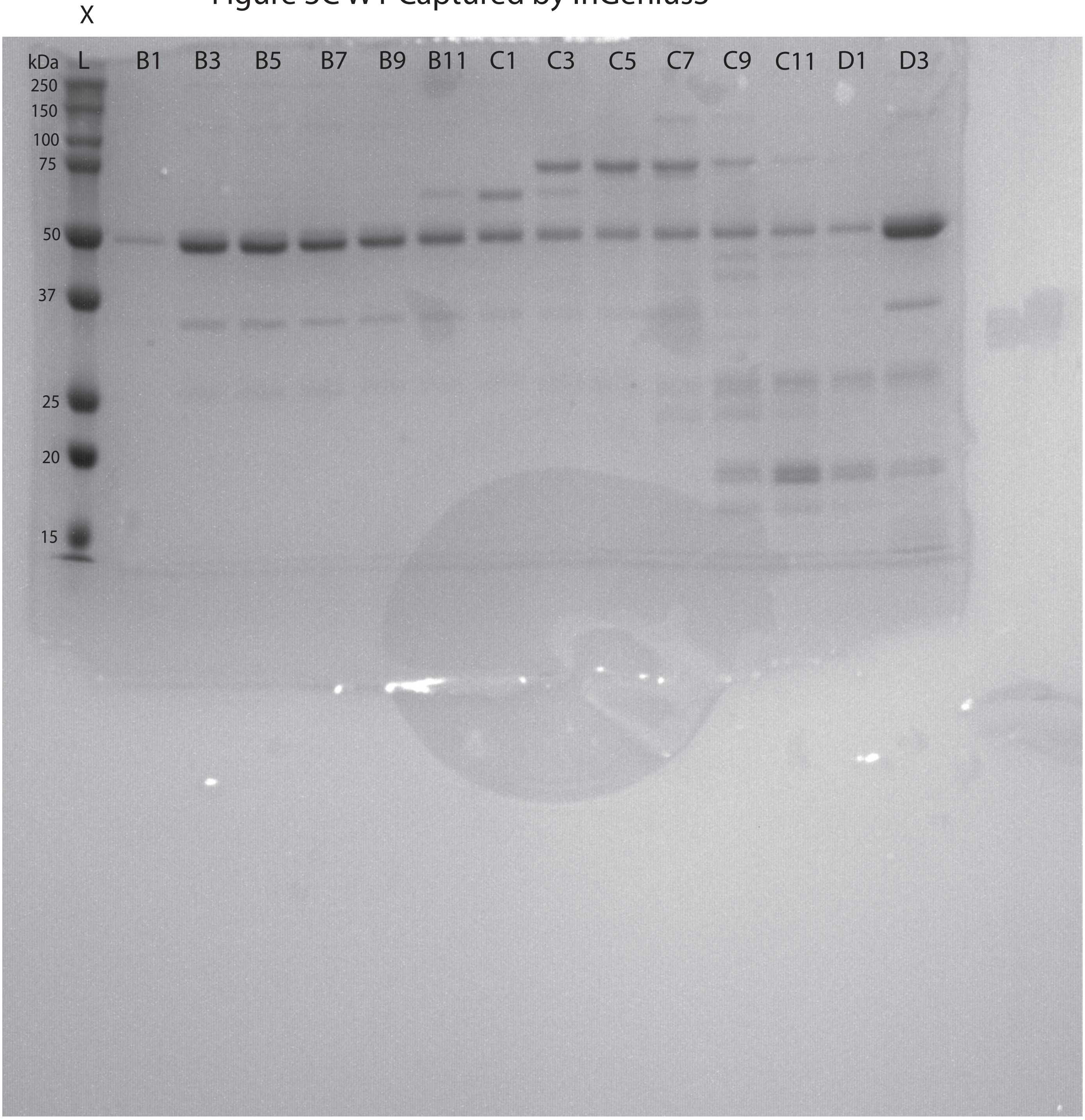

Figure 3C E149R-N150A Captured by InGenius3

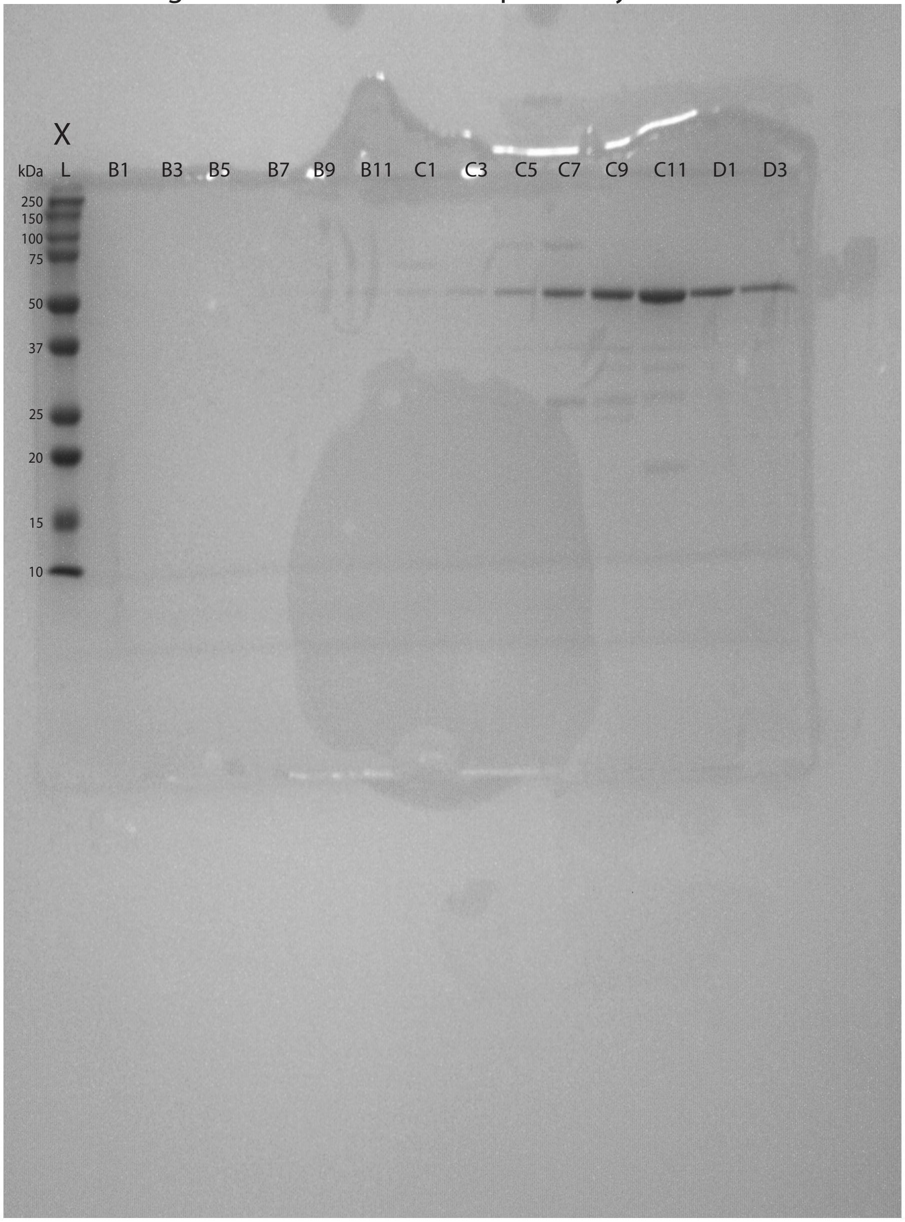

Figure 3C N364A-E365A Captured by InGenius3

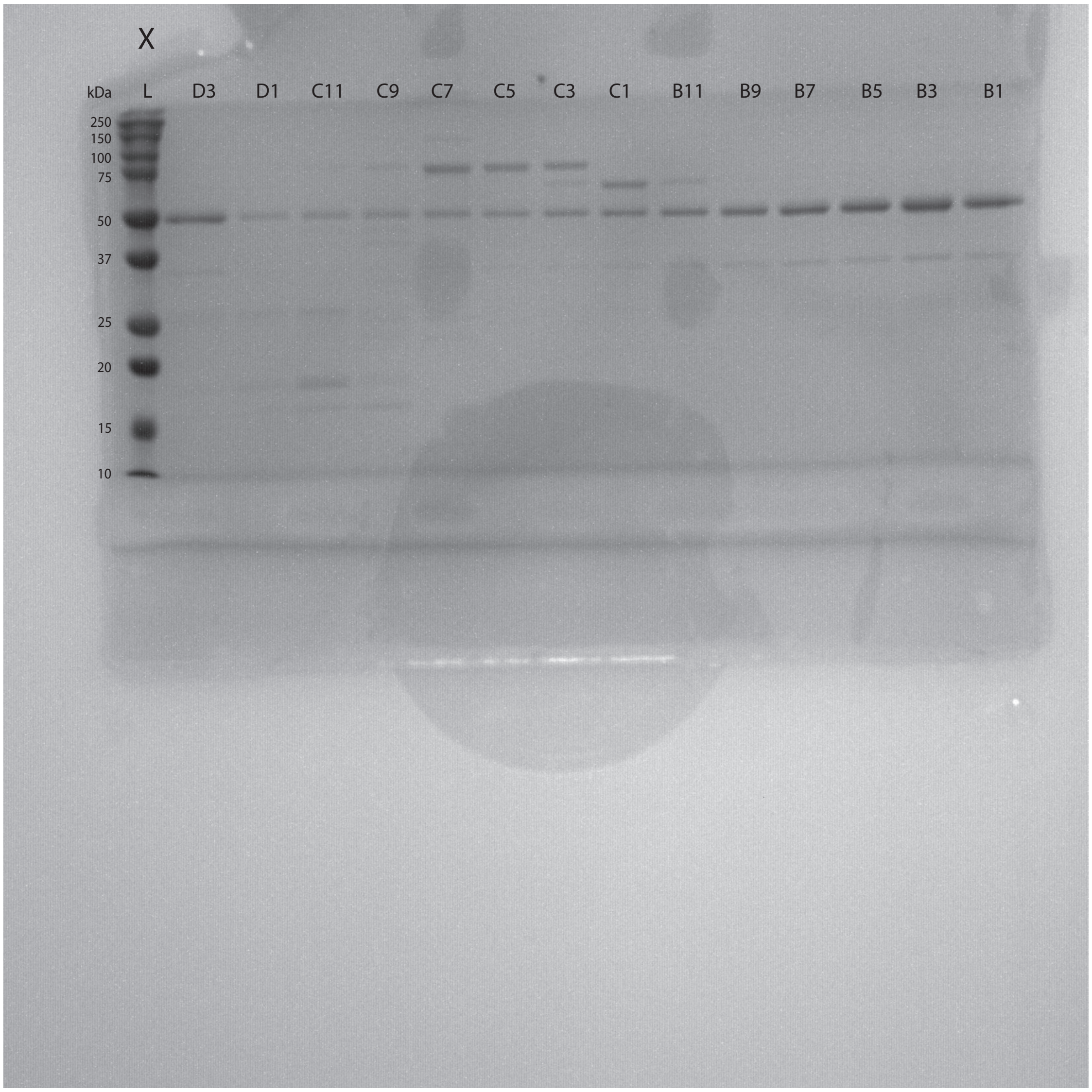

Note: gel image was flipped horizontally in Figure 3C of the manusript to stay consistent with the order of fractions and gels for all structural/interface mutants. The order the gel was loaded was fractions B1-D3 and then the standard protein ladder was added in the 15th well versus the 1st well.

Figure 3C I368A-N379A-R371E Captured by InGenius3

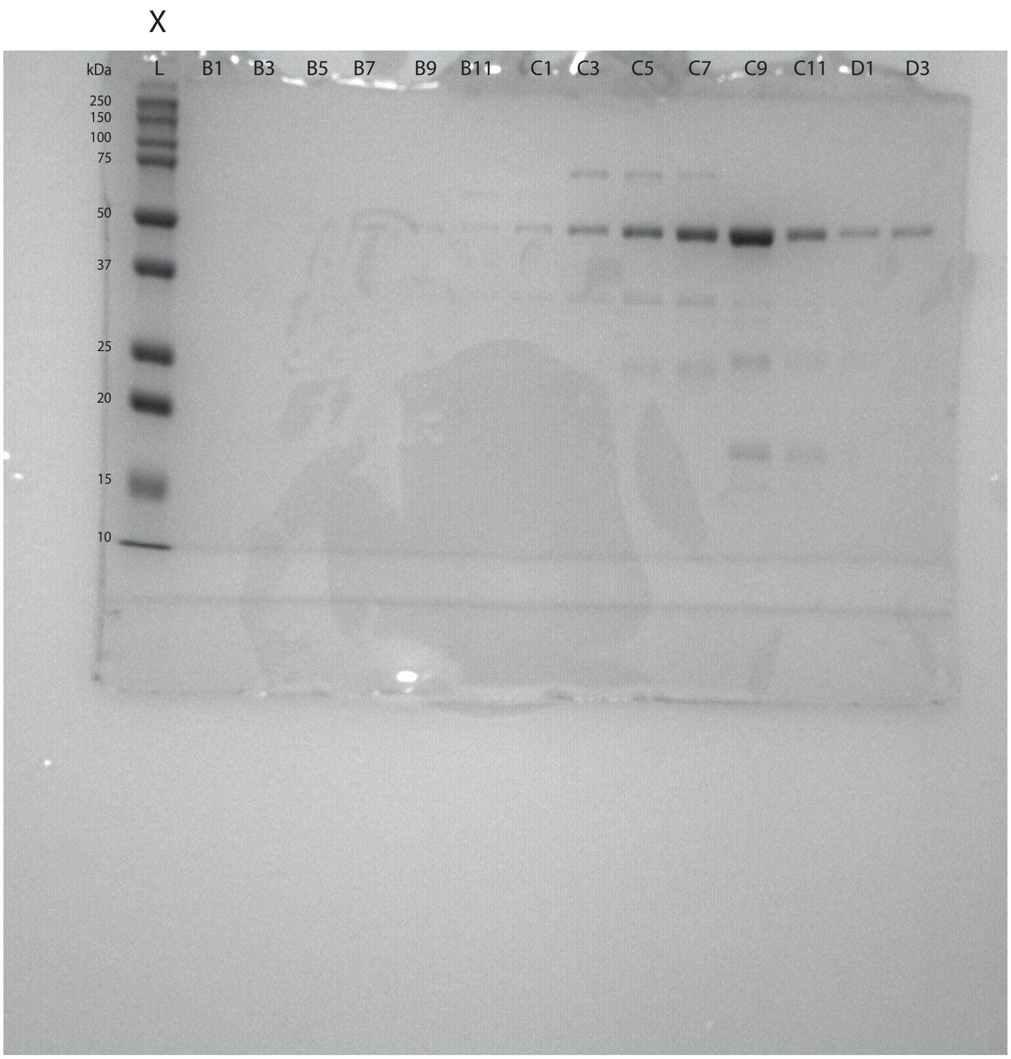

Figure 4D WT Captured by InGenius3

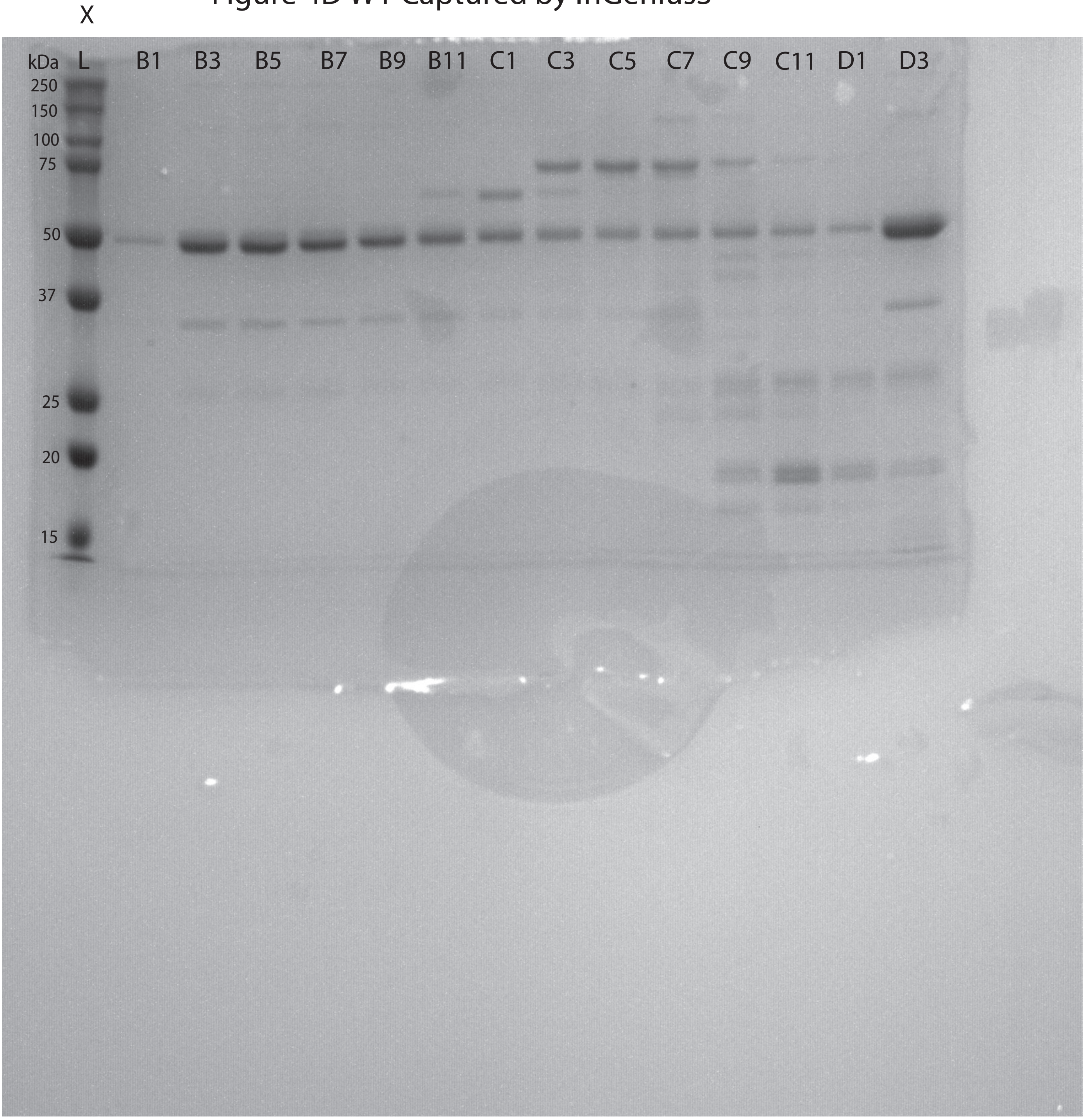

Figure 4D R117E Captured by InGenius3

X

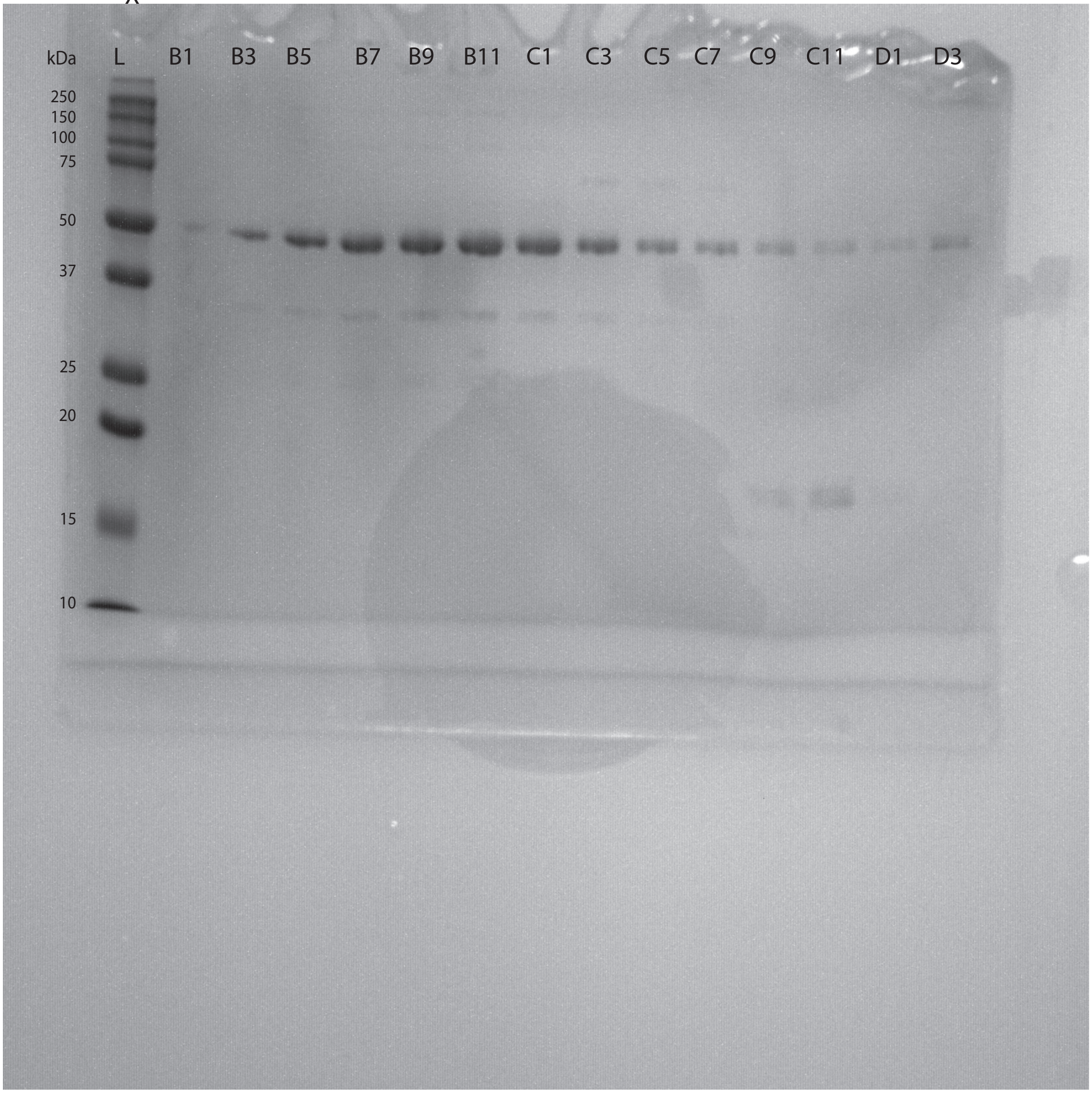

Figure 4D R151E Captured by InGenius3

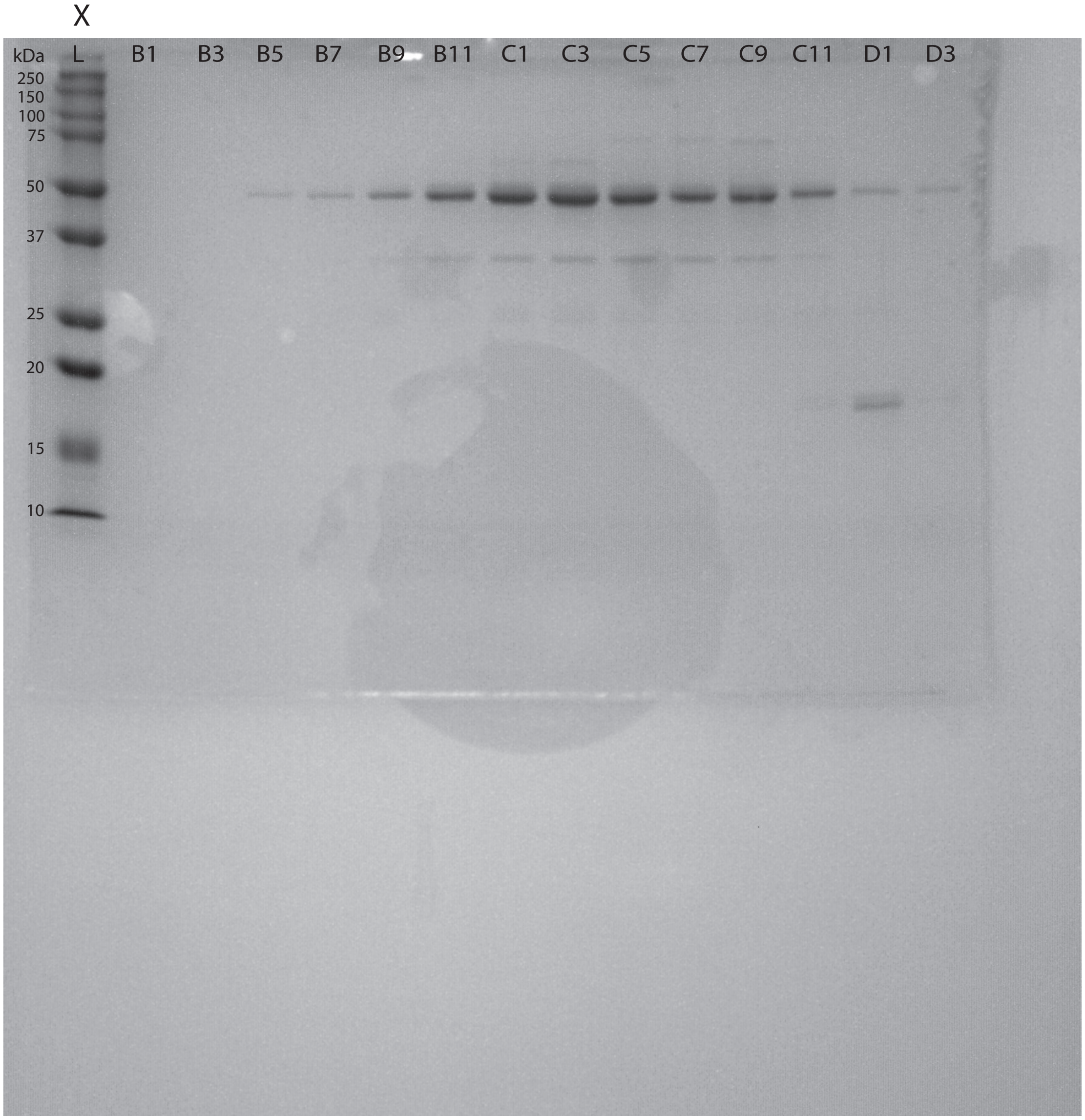

Figure 4D H352E Captured by InGenius3

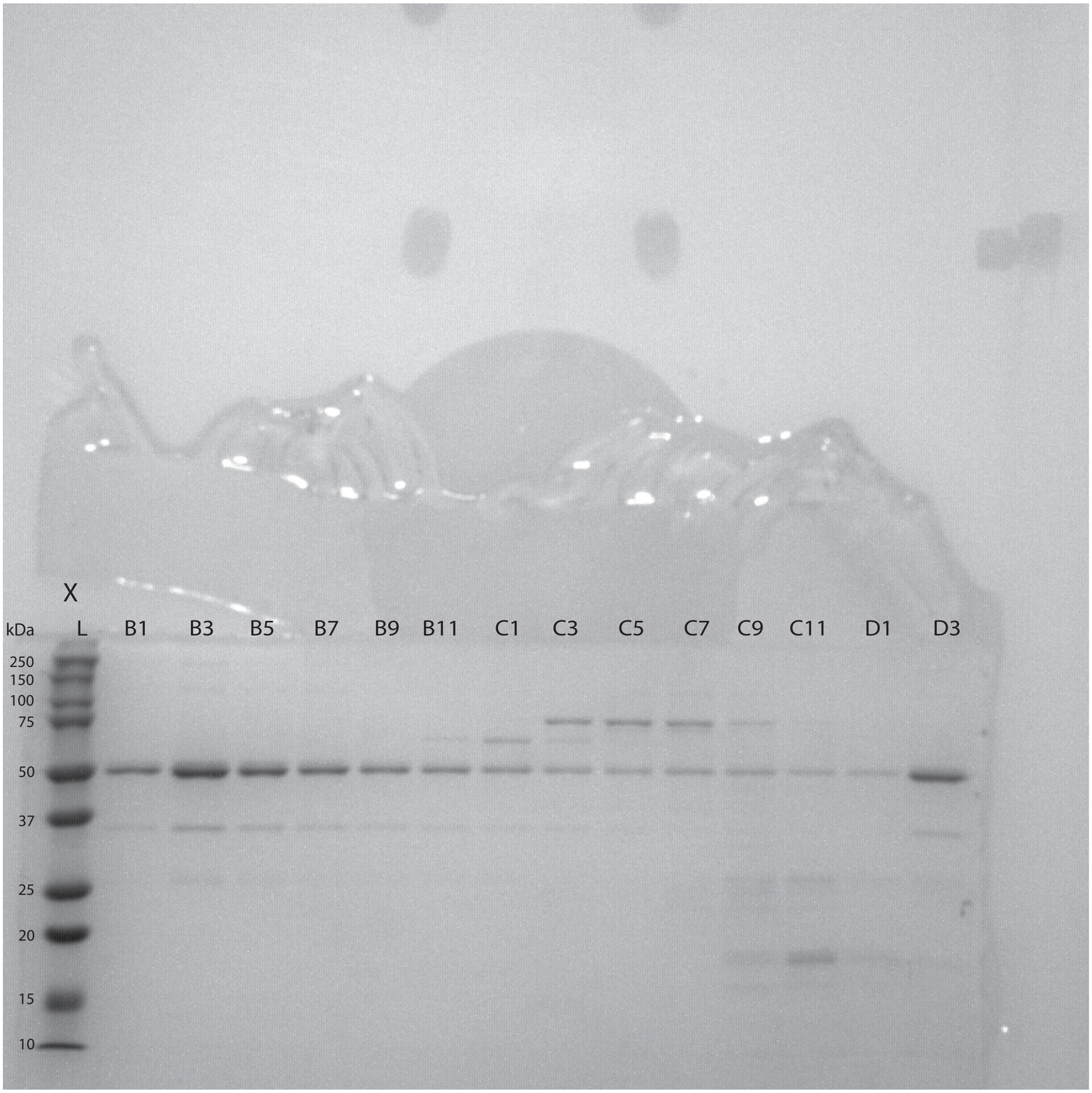

Figure 4D D355K Captured by InGenius3

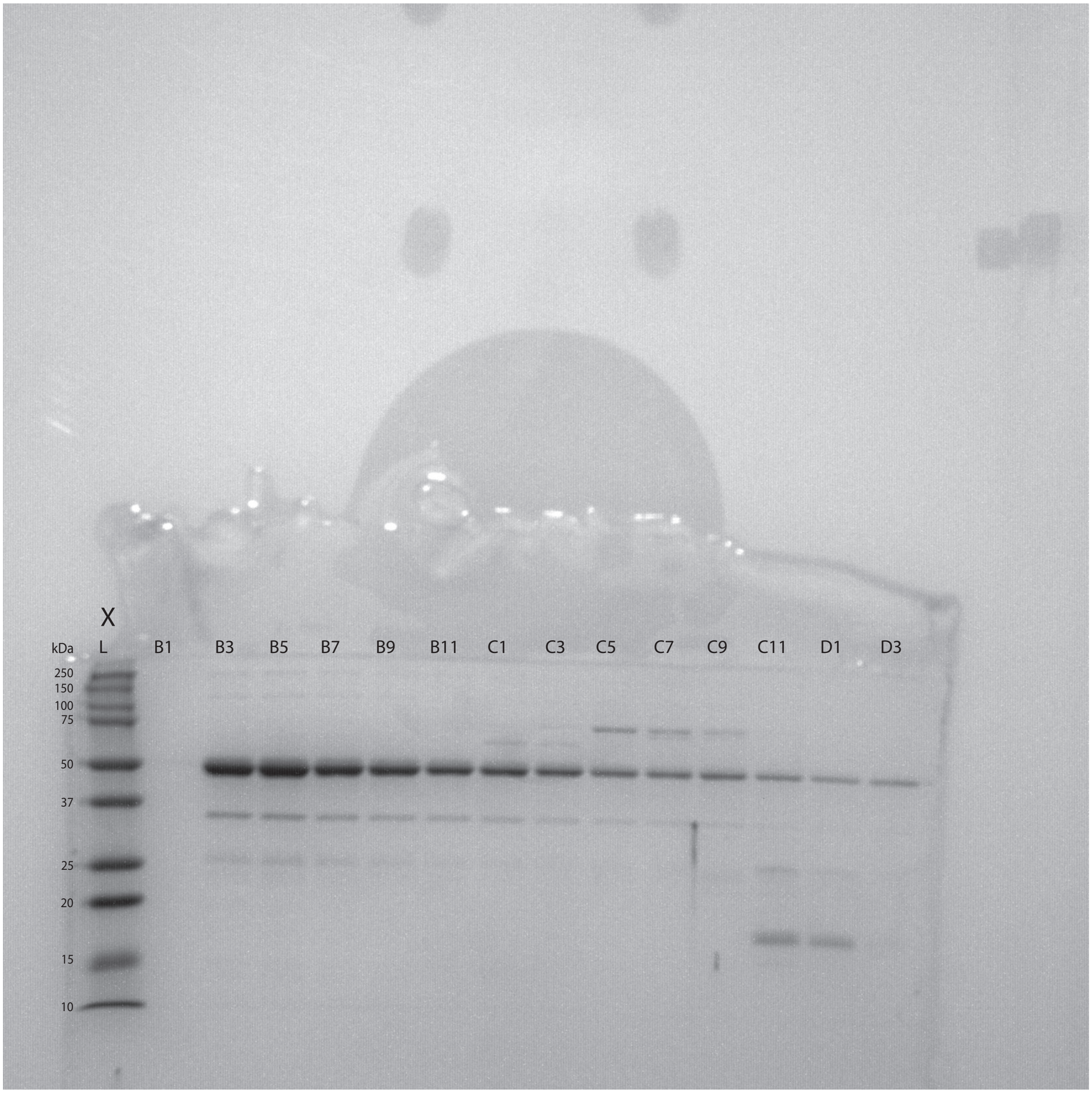

Figure 4D Y377K Captured by InGenius3

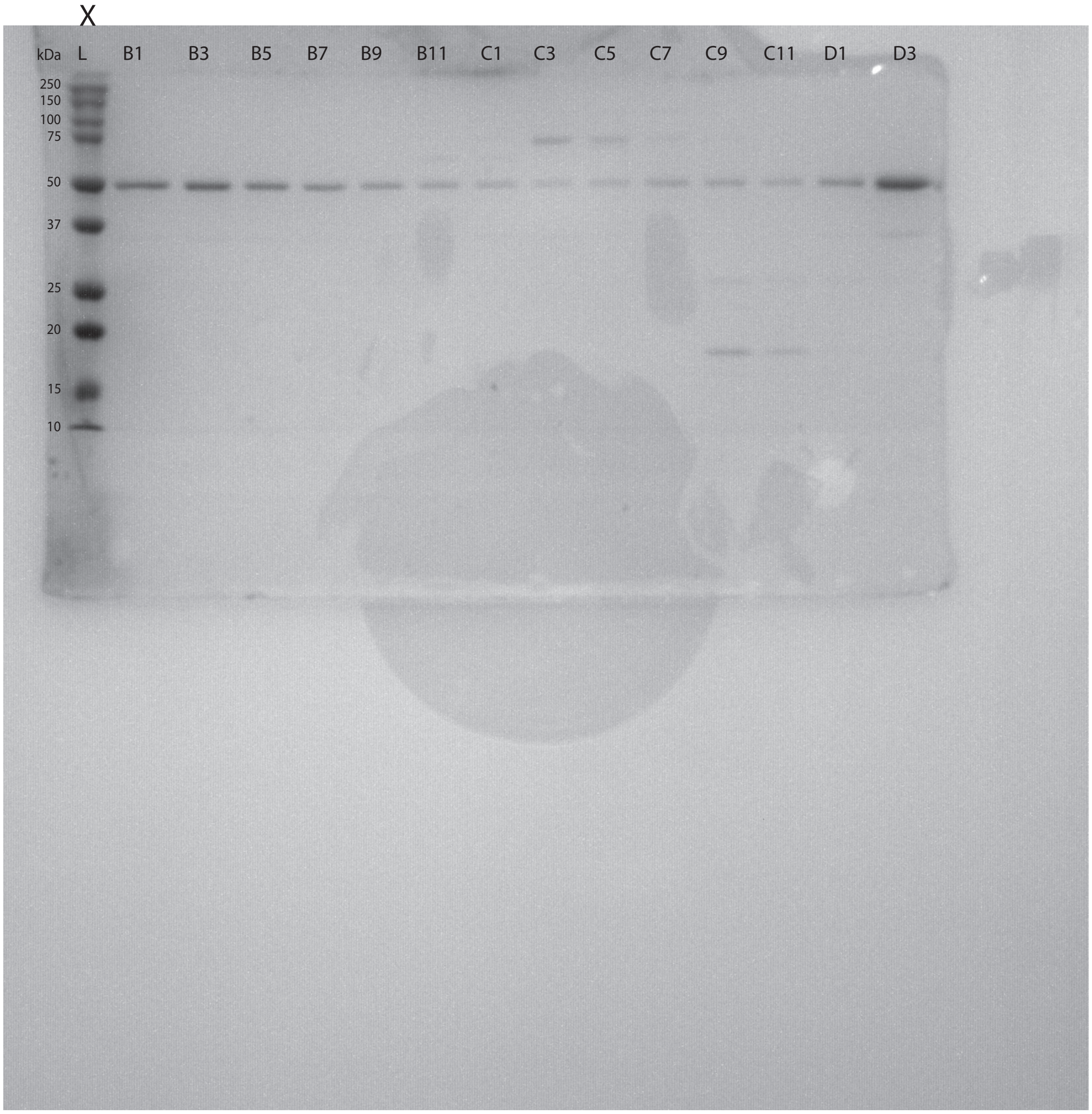

Figure 4D ΔK470-Q479 Captured by InGenius3

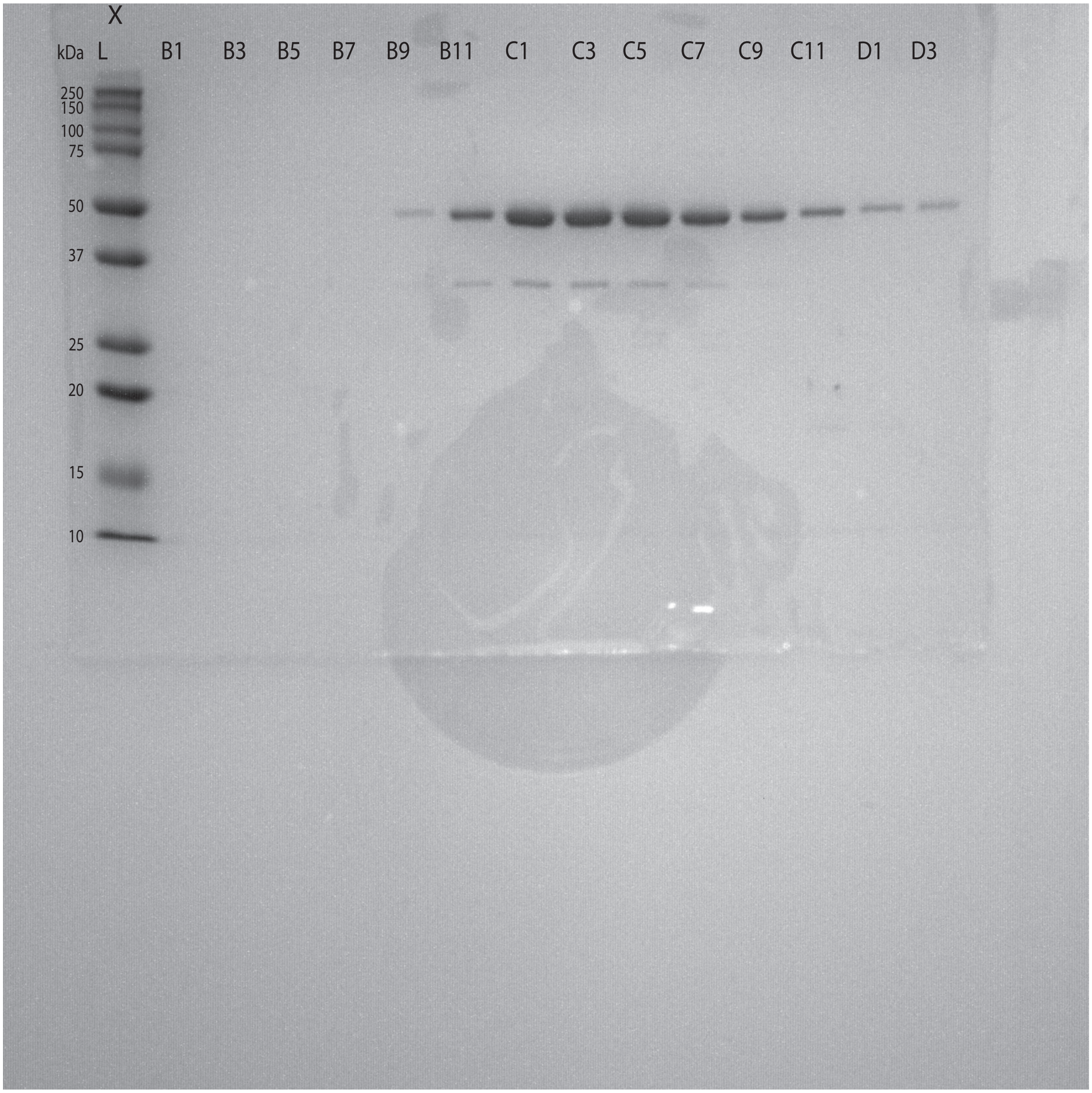

Figure 4D G474Y Captured by InGenius3

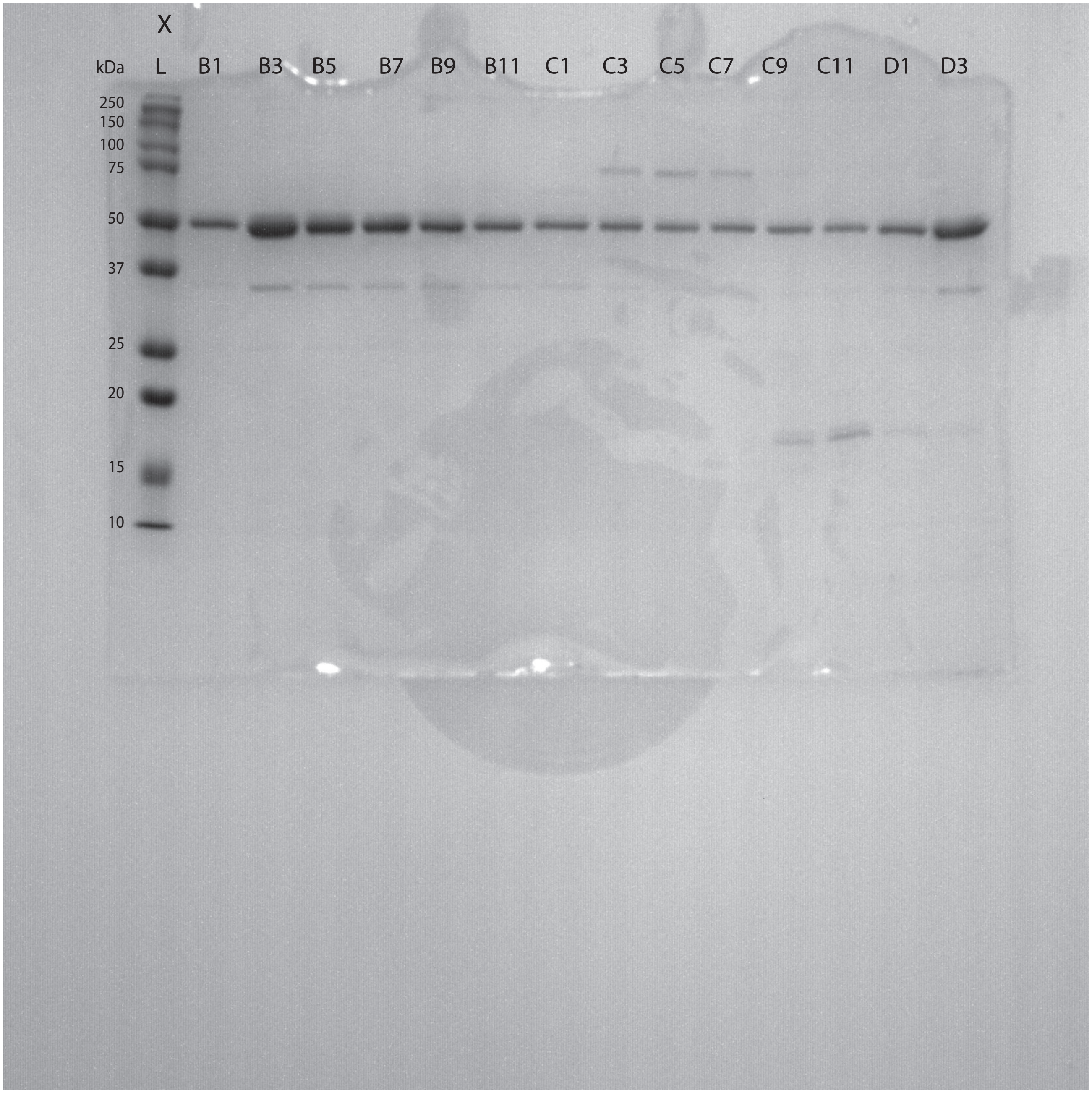

Figure 4D R477E Captured by InGenius3

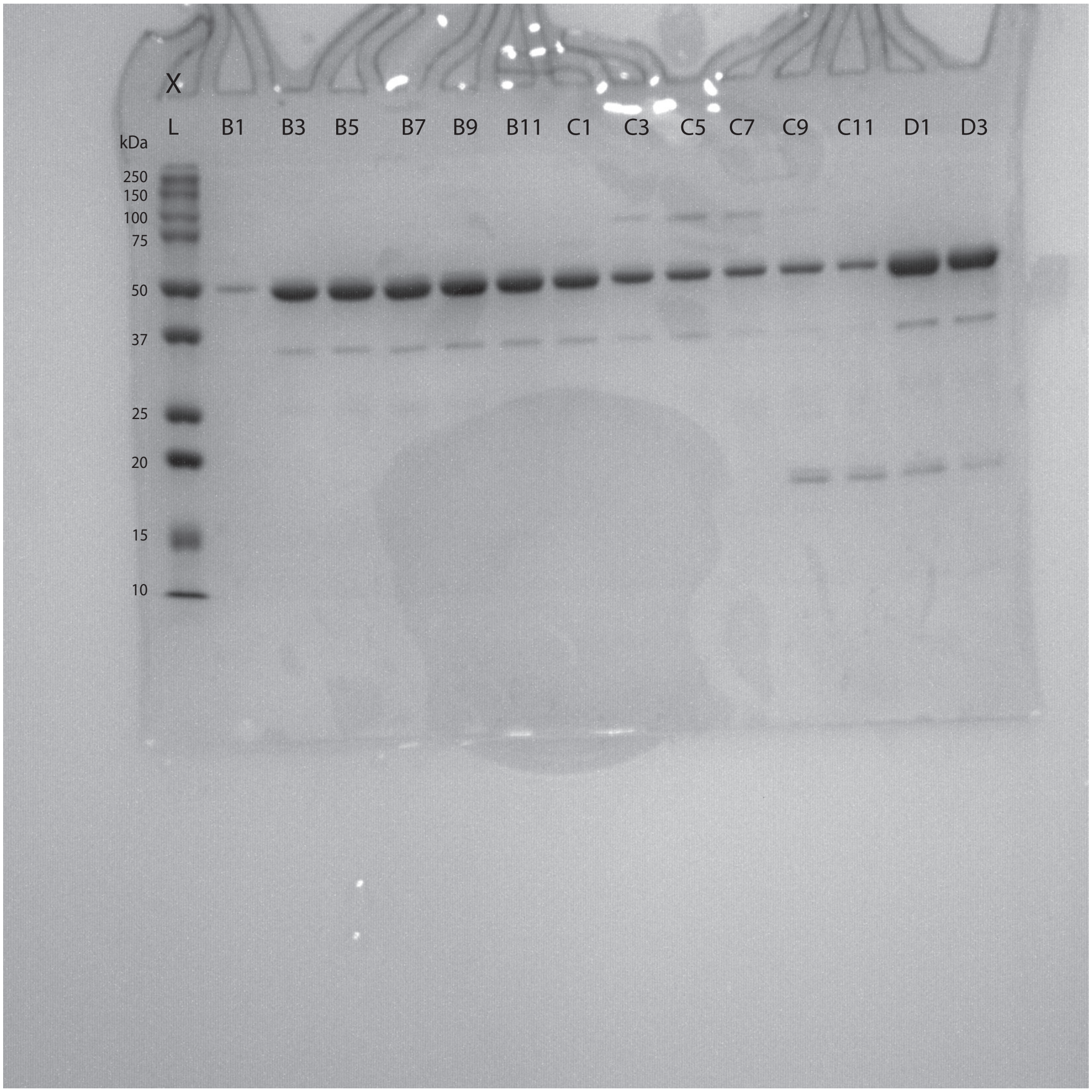

Figure S6B Captured by InGenius3

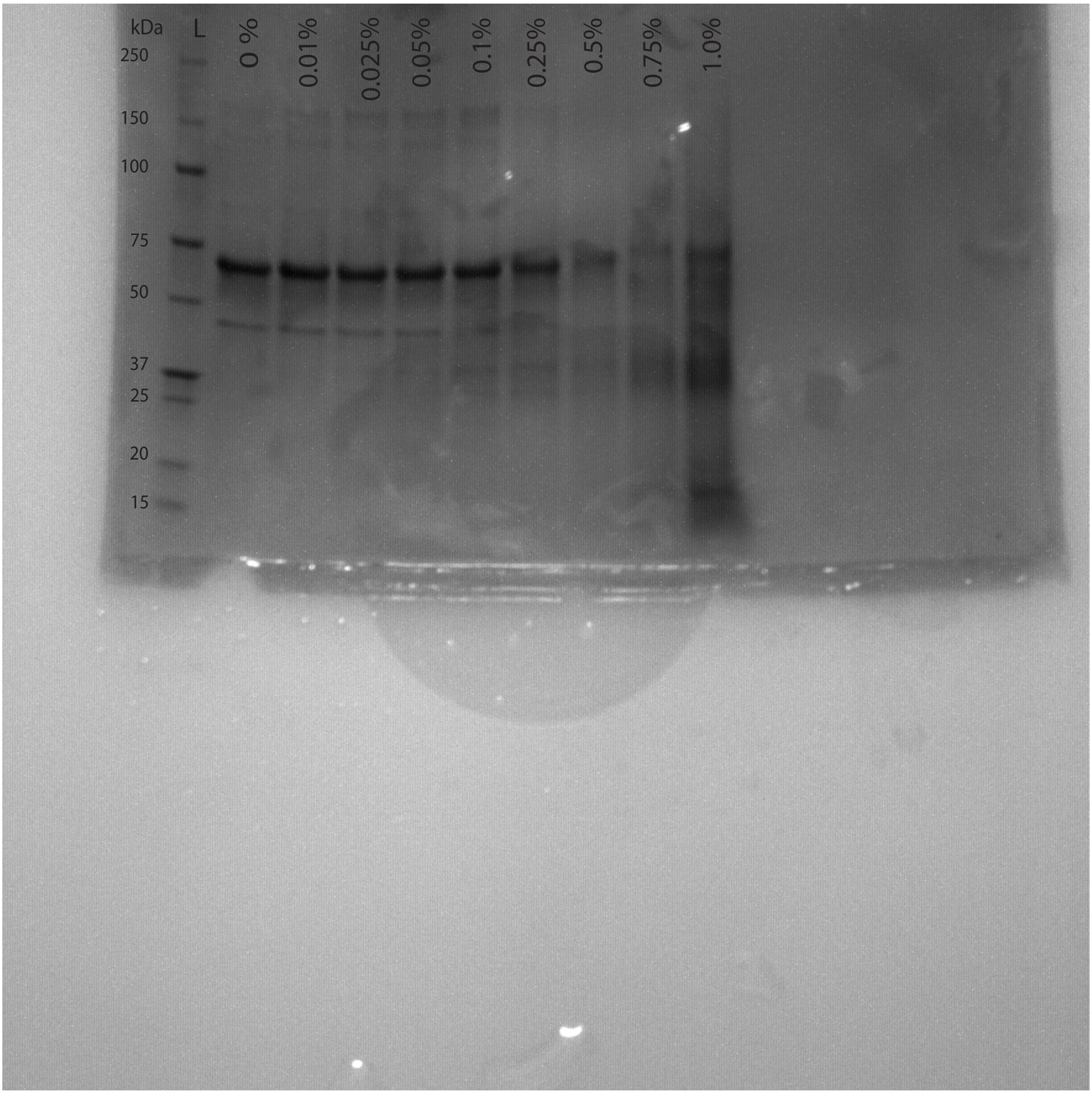

Figure S8A WT Captured by InGenius3

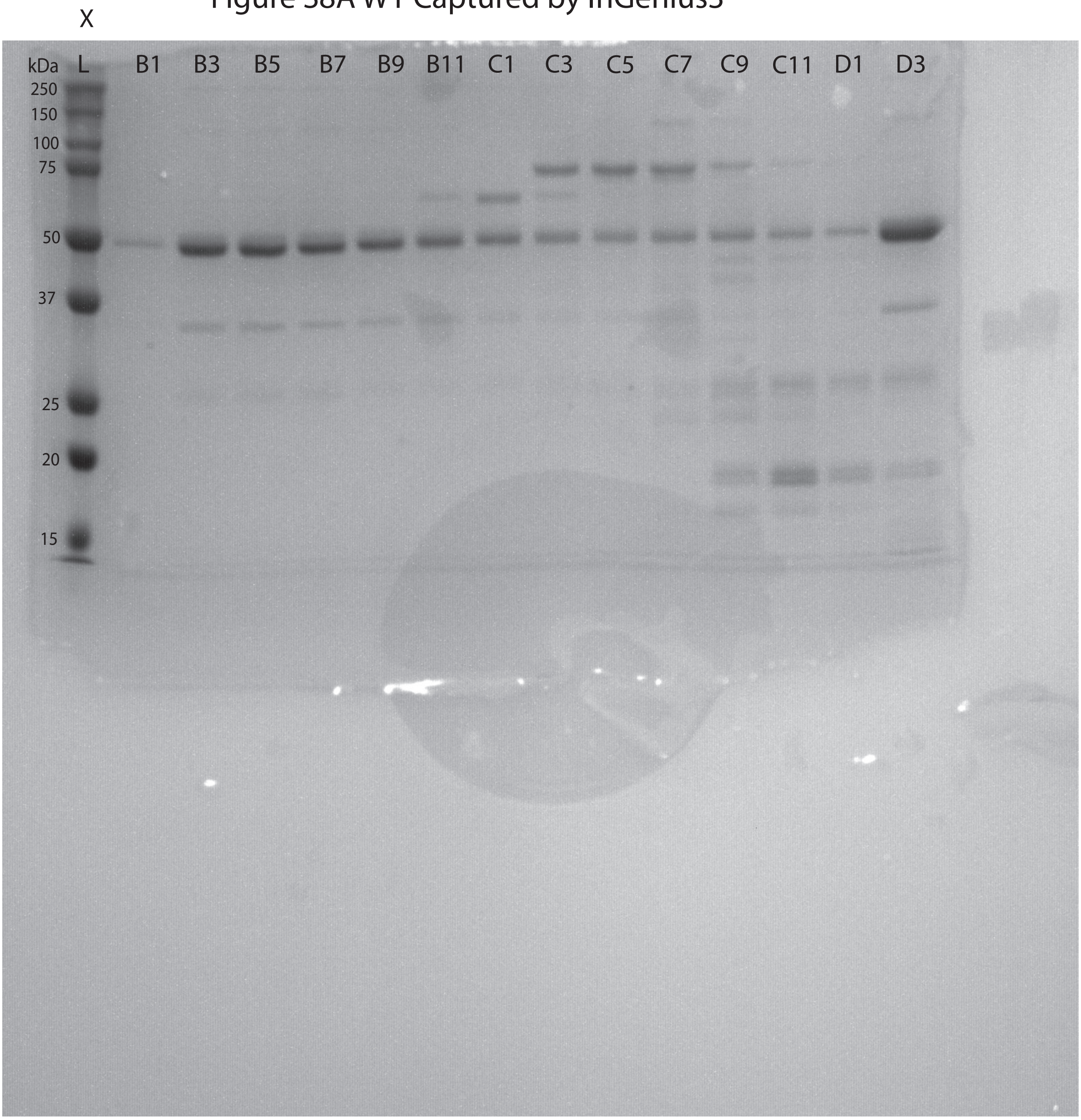

Figure S8A K274-K284 Mut Captured by BioRad GelDoc GO

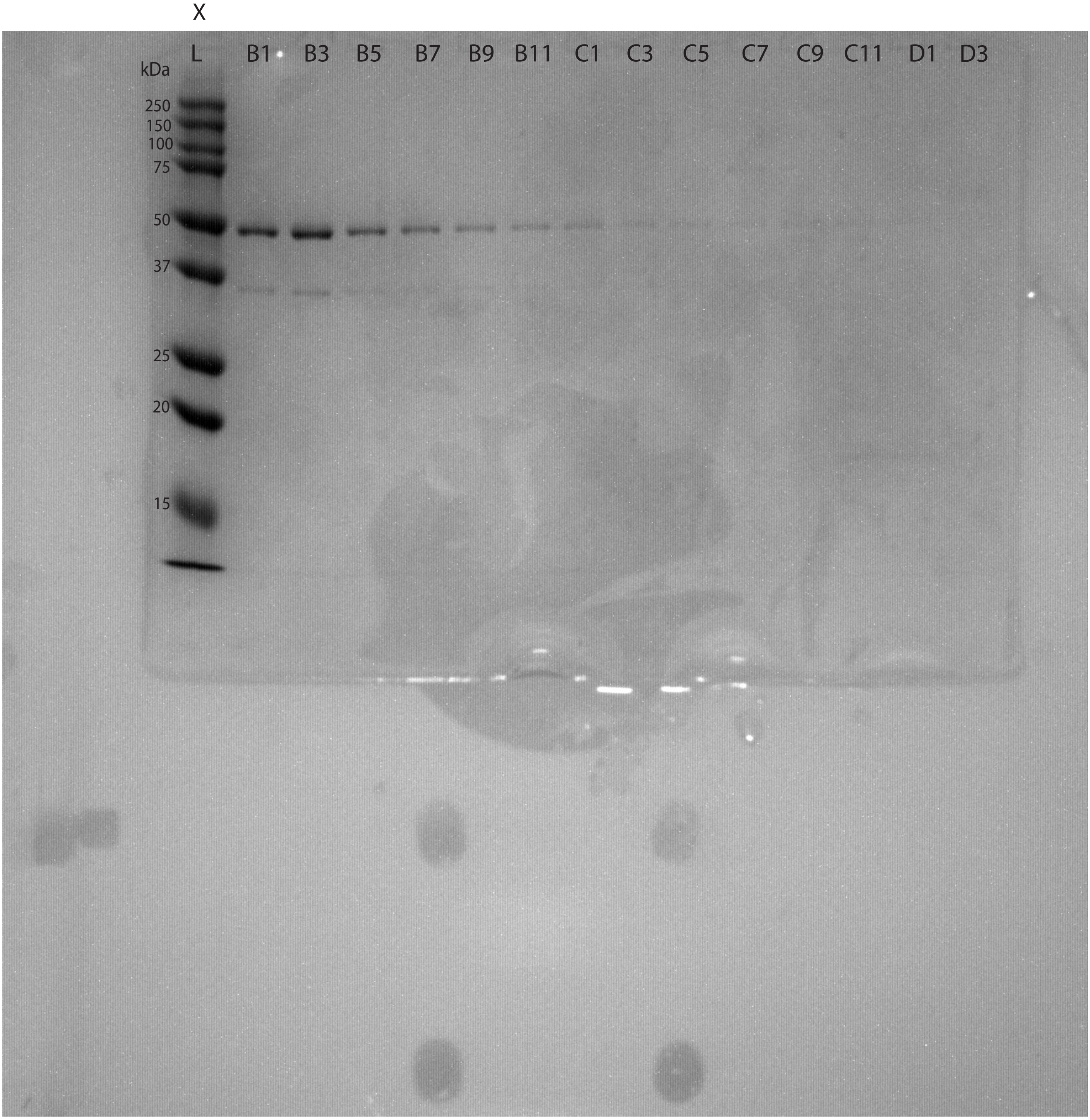

Figure S8A K259-K66 Mut Captured by BioRad GelDoc GO

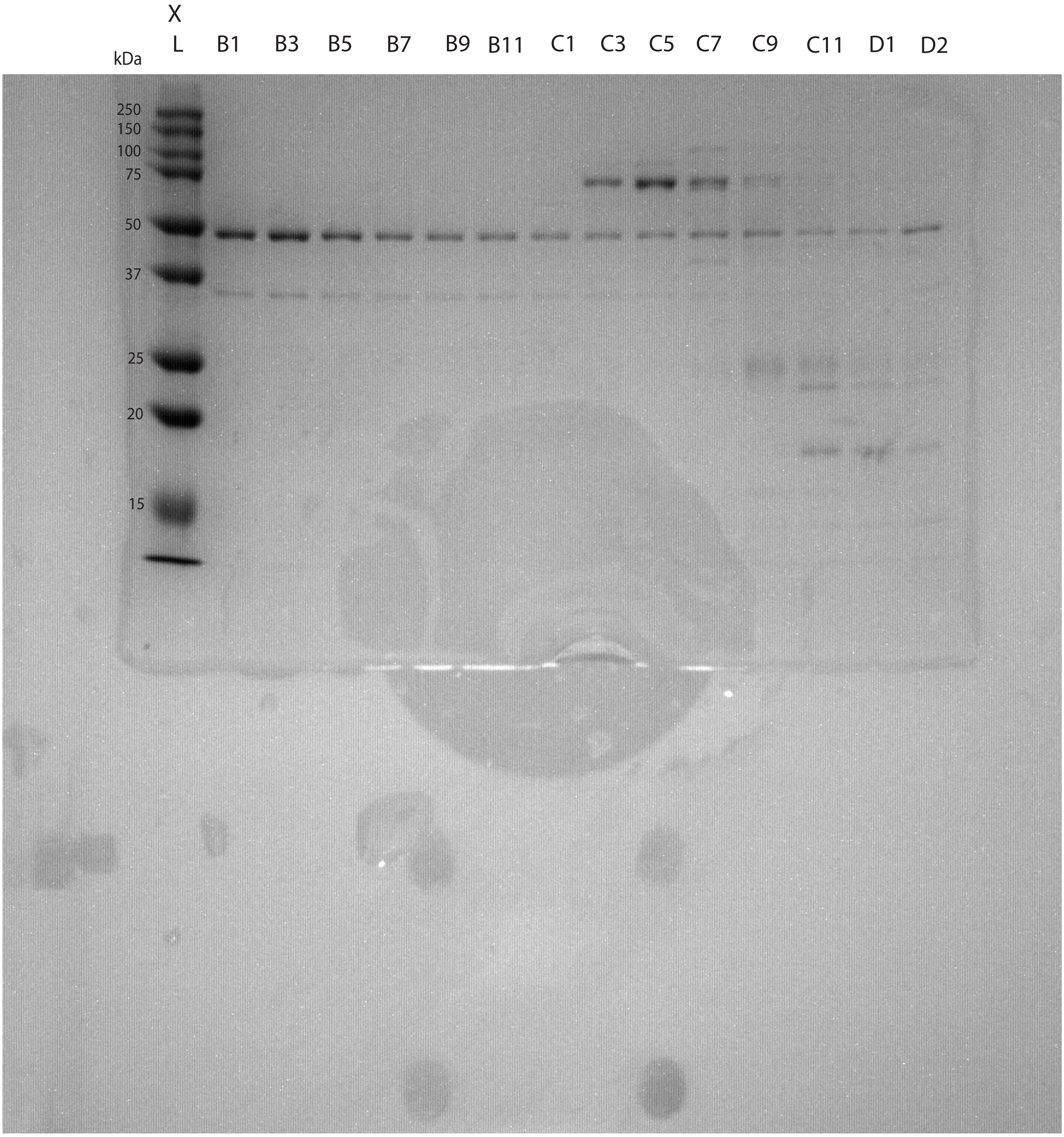

Figure S8A ΔL6 Captured by BioRad GelDoc GO

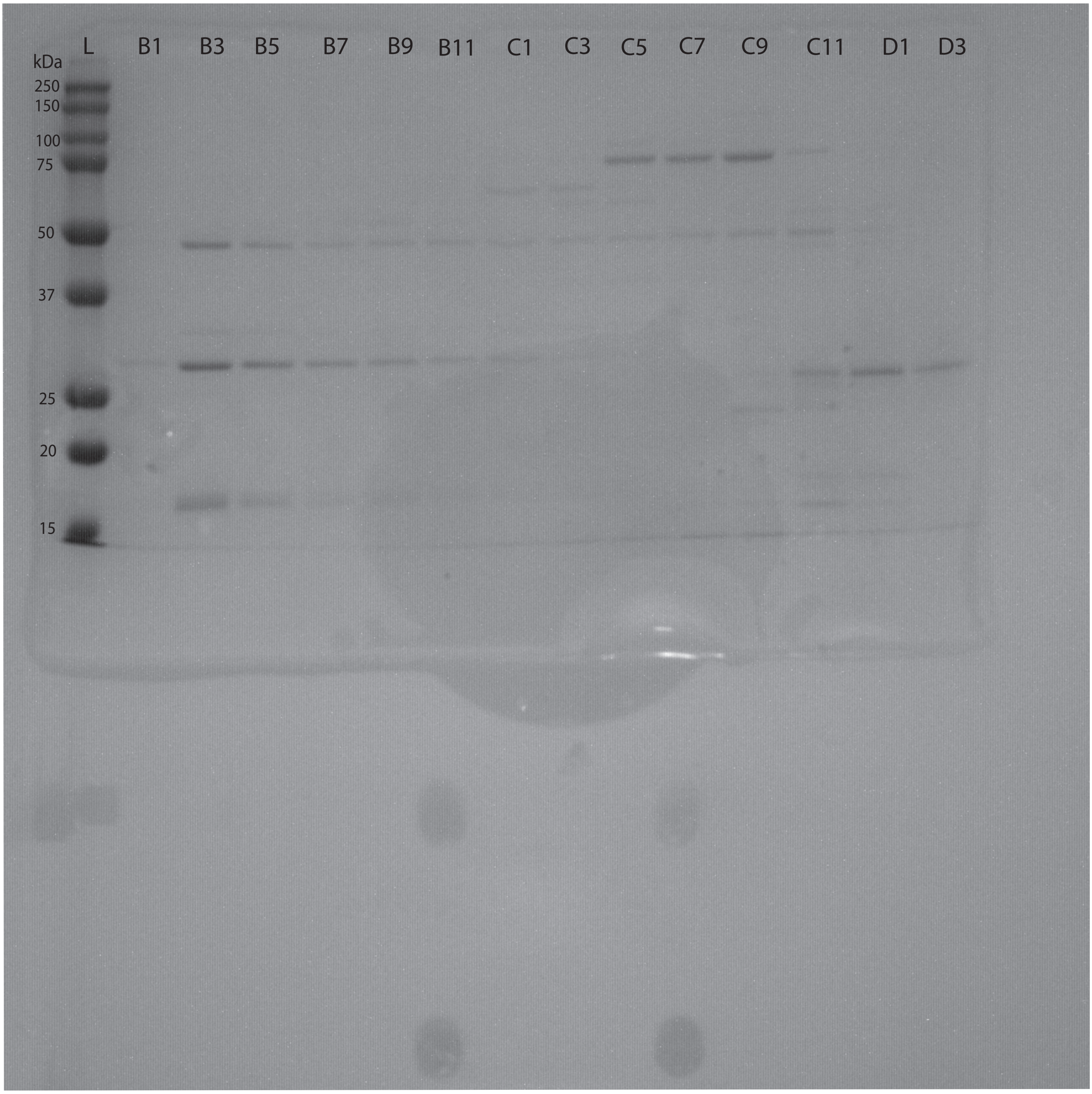

Supplement: S1 Raw images — Contains raw images for Figs 1C, 3C, 4D, S6B and S8A. (PDF) [file pbio.3001899.s014.pdf]
